# Sex-Dependent Synaptic Remodeling of the Somatosensory Cortex in Mice With Prenatal Methadone Exposure

**DOI:** 10.3389/adar.2022.10400

**Published:** 2022-04-25

**Authors:** Gregory G. Grecco, Jui Yen Huang, Braulio Muñoz, Emma H. Doud, Caliel D. Hines, Yong Gao, Brooke Rodriguez, Amber L. Mosley, Hui-Chen Lu, Brady K. Atwood

**Affiliations:** ^1^ Department of Pharmacology and Toxicology, School of Medicine, Indiana University, Indianapolis, IN, United States; ^2^ Medical Scientist Training Program, School of Medicine, Indiana University, Indianapolis, IN, United States; ^3^ The Linda and Jack Gill Center for Biomolecular Sciences, Indiana University, Bloomington, IN, United States; ^4^ Program in Neuroscience and Department of Psychological and Brain Sciences, Indiana University, Bloomington, IN, United States; ^5^ Department of Biochemistry and Molecular Biology, School of Medicine, Indiana University, Indianapolis, IN, United States; ^6^ Stark Neurosciences Research Institute, School of Medicine, Indiana University, Indianapolis, IN, United States

**Keywords:** methadone, prenatal opioid exposure, somatosensory cortex, proteomics, neurodevelopment

## Abstract

Rising opioid use among pregnant women has led to a growing population of neonates exposed to opioids during the prenatal period, but how opioids affect the developing brain remains to be fully understood. Animal models of prenatal opioid exposure have discovered deficits in somatosensory behavioral development that persist into adolescence suggesting opioid exposure induces long lasting neuroadaptations on somatosensory circuitry such as the primary somatosensory cortex (S1). Using a mouse model of prenatal methadone exposure (PME) that displays delays in somatosensory milestone development, we performed an un-biased multi-omics analysis and investigated synaptic functioning in the primary somatosensory cortex (S1), where touch and pain sensory inputs are received in the brain, of early adolescent PME offspring. PME was associated with numerous changes in protein and phosphopeptide abundances that differed considerably between sexes in the S1. Although prominent sex effects were discovered in the multi-omics assessment, functional enrichment analyses revealed the protein and phosphopeptide differences were associated with synapse-related cellular components and synaptic signaling-related biological processes, regardless of sex. Immunohistochemical analysis identified diminished GABAergic synapses in both layer 2/3 and 4 of PME offspring. These immunohistochemical and proteomic alterations were associated with functional consequences as layer 2/3 pyramidal neurons revealed reduced amplitudes and a lengthened decay constant of inhibitory postsynaptic currents. Lastly, in addition to reduced cortical thickness of the S1, cell-type marker analysis revealed reduced microglia density in the upper layer of the S1 that was primarily driven by PME females. Taken together, our studies show the lasting changes on synaptic function and microglia in S1 cortex caused by PME in a sex-dependent manner.

## Introduction

Despite efforts to curtail the opioid addiction crisis, opioid use and misuse continue to represent a major health concern. As the crisis has further developed, opioid-exposed infants have emerged as a particularly vulnerable population that is relatively understudied. A significant rise in maternal opioid use disorder (OUD) at delivery has translated into a substantial increase in neonatal opioid withdrawal syndrome (NOWS) by 3.3 per 1000 births representing an 82% increase in NOWS between 2010 and 2017 (1). Indeed, nearly half of all states within the US witnessed at least a 100% increase in both NOWS and maternal OUD with some states seeing a nearly fourfold increase in NOWS rates during this time period (1). Although often complicated by significant variations in prenatal/postnatal environment, prenatal opioid exposure is associated with numerous physical and developmental impairments including poorer outcomes at birth and deficits in attention, behavioral regulation, motor skills, and cognitive performance throughout early childhood development (2–4).

In an effort to advance our understanding of the clinical implications of prenatal opioid exposure, there has been a growing interest in developing preclinical models of prenatal opioid exposure (5, 6). These animal models have generally recapitulated findings described in clinical studies with prenatal opioid exposed animals demonstrating hyperactivity (7), cognitive dysfunction (8, 9), and delayed neurodevelopment (10, 11). To better replicate epidemiological trends in maternal opioid use (12, 13), our laboratory developed a mouse model of prenatal methadone exposure (PME) as this recapitulates the growing proportion of prenatal opioid exposed cases resulting from treatment of OUD in reproductive age women (14). Rodent pups with PME exhibited withdrawal-like symptoms at birth, reduced growth, and altered behavior in an open field when repeatedly assessed throughout the weaning period (14). Additionally, several developmental milestones of sensorimotor-based behaviors were delayed in PME offspring including cliff aversion, surface righting, and the forelimb grasp task indicating offspring may struggle to translate multimodal sensory input into motor behaviors (14). Indeed, we discovered motor neurons of the primary motor cortex exhibited alterations in sub-threshold firing properties and local circuitry associated with this aberrant behavioral development of PME mice (14).

The maladaptive development of somatosensory circuitry may contribute to the sensorimotor behavioral phenotype of these PME mice. For instance, tactile information via whisker stimulation is necessary for the display of some developmental milestones such as the cliff aversion (15). These findings indicate the somatosensory system, specifically the primary somatosensory cortex (S1), may be an integral component of the neural circuit controlling reflexive behaviors during early development. To determine if pathological adaptations exist in the S1 of our PME model that may contribute to the impaired sensorimotor behavioral development (14), we performed quantitative global proteomics and phosphoproteomics of the S1 alongside electrophysiological and neuroanatomical assessments of the S1 excitatory and inhibitory synapses in early adolescent PME and prenatal saline exposed (PSE) offspring.

## Materials and Methods

### Animals and Model Generation

Protocols were approved by the Indiana University School of Medicine Institutional Animal Care and Use Committee and guidelines established by the National Institutes of Health were used to conduct animal care and research. An extensive description and characterization of model generation have been published elsewhere (14). Female C57BL/6J mice were randomly assigned to receive either saline (10 mL/kg) or oxycodone treatments to model oxycodone dependence prior to initiating treatment for OUD. We have previously demonstrated this oxycodone dosing strategy induces robust opioid dependency (14). All saline or oxycodone doses were administered subcutaneously twice daily at least 7 hours apart. Following 9 days of oxycodone injections, oxycodone-dependent mice began receiving methadone (10 mg/kg s.c. b.i.d.) while saline-treated animals continued to receive saline injections. Five days following the start of methadone treatment, an 8-week-old C57BL/6J male mouse was placed into the cage of each female for 4 days. Methadone or saline treatments continued throughout the remainder of pregnancy and postnatal period up to weaning. We previously demonstrated that this dose of methadone leads to plasma levels within the therapeutic range and produces dependency in both dams and offspring (14). Additionally, we find this dosing strategy only minimally impacts pregnancy characteristics and does not influence maternal care (14). Oxycodone and methadone were obtained from the National Institute on Drug Abuse Drug Supply Program. Both offspring in our previous study and the current one were weaned at approximately 3 weeks of age and group housed (3–5 per cage). Early adolescent offspring (P21–P36) were used for the proteomics, immunohistochemical, and electrophysiological studies described here.

### Proteomics and Phosphoproteomics

#### Protein Preparation

Sample preparation, mass spectrometry analysis, bioinformatics, and data evaluation for quantitative proteomics and phosphoproteomics experiments were performed in collaboration with the Indiana University Proteomics Core similar to our previous studies (16).

Animals were rapidly decapitated without anesthesia between 1 p.m. and 4 p.m. by a blinded researcher and tissue was dissected bilaterally. Slices were cut in a 0.5 mm coronal mouse brain matrix and whole S1 was carefully dissected from each slice. Tissue was immediately snap frozen in isopentane on dry ice and stored until later processing. Flash frozen brain lysates were homogenized using a BeadBug™ 6 (Benchmark scientific Cat No: D1036, 3 mm zirconium beads Cat No: D1032-30, 10 rounds of 30 × 30 s,4°C) in 1 mL of 8 M urea (CHEBI: 16199) in 100 mM Tris, pH 8.5 (CHEBI: 9754). Samples were next sonicated on a Bioruptor^®^ sonication system (Diagenode Inc. United States, North America cat number B01020001) with 30 s/30 s on/off cycles for 15 min in a water bath at 4°C. After subsequent centrifugation at 14,000 rcf for 20 min, protein concentrations were determined by Bradford protein assay (BioRad Cat No: 5000006). 100 µg equivalent of protein from each sample were then treated with 5 mM tris(2-carboxyethyl)phosphine hydrochloride (Sigma-Aldrich Cat No: C4706) to reduce disulfide bonds and the resulting free cysteine thiols were alkylated with 10 mM chloroacetamide (Sigma Aldrich Cat No: C0267). Samples were diluted with 50 mM Tris.HCl pH 8.5 (Sigma-Aldrich Cat No: 10812846001) to a final urea concentration of 2 M for overnight Trypsin/Lys-C digestion at 35°C (1:100 protease:substrate ratio, Mass Spectrometry grade, Promega Corporation, Cat No: V5072.) (17, 18).

#### Peptide Purification and Labeling

Digestion was halted by addition of 0.3% v/v trifluoroacetic acid (TFA), and peptides were desalted on Waters Sep-Pak^®^ Vac cartridges (Waters™ Cat No: WAT054955) with a wash of 1 mL 0.1% TFA followed by elution in 0.6 mL of 70% acetonitrile 0.1% formic acid (FA). Peptides were dried by speed vacuum and resuspended 50 mM triethylammonium bicarbonate. Peptide concentrations were checked by Pierce Quantitative colorimetric assay (Cat No: 23275). The same amount of peptide from each sample was then labeled for 2 hours at room temperature, with 0.5 mg of Tandem Mass Tag Pro (TMTpro) reagent (16-plex kit, manufactures instructions Thermo Fisher Scientific, TMTpro™ Isobaric Label Reagent Set; Cat No: 44520, lot no. VI310352) (18). Labelling reactions were quenched with 0.3% hydroxylamine (v/v) at room temperature for 15 min. Labeled peptides were then mixed and dried by speed vacuum. The TMT-labeled peptide mix was desalted to remove excess label using a 100 mg Waters SepPak cartridge, eluted in 70% acetonitrile, 0.1% formic acid and lyophilized to dryness.

#### Phosphopeptide Enrichment

Phosphopeptides were enriched from the mixed, labeled peptides on one spin tip from a High-Select™ TiO2 Phosphopeptide Enrichment Kit (capacity of 1–3 mg; Thermo Fisher Scientific, catalog A32993). After preparing spin tips, labeled and mixed peptides were repeatedly applied to the TiO2 spin tip, eluted and immediately dried as per manufacturer’s instructions. Prior to LC/MS/MS the phosphopeptides were resuspended in 25 µL 0.1% formic acid. The flow through from each tip was saved for global proteomics.

#### High pH Basic Fractionation

The flowthrough and wash volumes from phosphoproteomic enrichment were lyophilized, resuspended in 150 uL 10 mM formate pH 10 and fractionated using an offline Thermo UltiMate 3000 HPLC with a Waters Xbridge C18 column (3.5 µm × 4.6 mm x 250 mm, pn 186003943; Buffer A: 10 mM formate pH 10, Buffer B: 10 mM formate pH 10, 95% acetonitrile, gradient 1 mL/min 0–15%B over 5 min, 15–20% B over 5 min, 20–35%B over 75 min, 35–50% B over 5 min, 50–60% B over 10 min and a 6 min hold at 60% B). Fractions were collected continuously every 60 s into 96 well plates. Initial and late fractions with minimal material were combined and lyophilized. The remaining fractions were concatenated into 24 fractions, dried down, and resuspended in 50 µL 0.1% FA prior to online LC-MS (19, 20).

#### Nano-LC-MS/MS Analysis

Nano-LC-MS/MS analyses were performed on an EASY-nLC™ HPLC system (SCR: 014993, Thermo Fisher Scientific) coupled to Orbitrap Fusion™ Lumos™ mass spectrometer (Thermo Fisher Scientific). One fifth of the phosphopeptides and one tenth of each global peptide fraction was loaded onto a reversed phase EasySprayTM C18 column (2 μm, 100 Å, 75 μm × 50 cm, Thermo Scientific Cat No: ES802A) at 400 nL/min. Peptides were eluted from 4 to 28% with mobile phase B [Mobile phases A: 0.1% FA, water; B: 0.1% FA, 80% Acetonitrile (Fisher Scientific Cat No: LS122500)] over 160 min; 28%–35% B over 5 min; 35–50% B for 14 min; and dropping from 50 to 10% B over the final 1 min. The mass spectrometer method was operated in positive ion mode with a 4 s cycle time data-dependent acquisition method with advanced peak determination and Easy-IC (internal calibrant). Precursor scans (m/z 400-1750) were done with an orbitrap resolution of 120,000, RF lens% 30, maximum inject time 50 ms, standard AGC target, including charges of 2–6 for fragmentation with 60 s dynamic exclusion. MS2 scans were performed with a fixed first mass of 100 m/z, 34% fixed CE, 50000 resolution, 20% normalized AGC target and dynamic maximum IT. The data were recorded using Thermo Fisher Scientific Xcalibur (4.3) software (Thermo Fisher Scientific Inc.).

#### Proteome and Phosphoproteome Data Processing

Resulting RAW files were analyzed in Proteome Discover™ 2.5 (Thermo Fisher Scientific, RRID: SCR_014477) with a mus musculus UniProt FASTA plus common contaminants. SEQUEST HT searches were conducted with a maximum number of 3 missed cleavages; precursor mass tolerance of 10 ppm; and a fragment mass tolerance of 0.02 Da. Static modifications used for the search were, 1) carbamidomethylation on cysteine (C) residues; 2) TMTpro label on lysine (K) residues and the N-termini of peptides. Dynamic modifications used for the search were TMTpro label on N-termini of peptides, oxidation of methionines, phosphorylation on serine, threonine or tyrosine, and acetylation, methionine loss or acetylation with methionine loss on protein N-termini. Percolator False Discovery Rate was set to a strict setting of 0.01 and a relaxed setting of 0.05. IMP-ptm-RS node was used for all modification site localization scores. Values from both unique and razor peptides were used for quantification. In the consensus workflows, peptides were normalized by total peptide amount with no scaling. Quantification methods utilized TMTpro isotopic impurity levels available from Thermo Fisher Scientific. Reporter ion quantification was allowed with S/N threshold of 7 and co-isolation threshold of 50%. Data shown is for PME/PSE abundance value ratios (AR). Resulting grouped abundance values for each sample type, AR values; and respective p-values (t-test) from Proteome Discover™ were exported to Microsoft Excel. Full datasets are provided in the Supplementary Material. The raw data files can be found here: https://github.com/gggrecco/S1-Omics.

### Electrophysiology

#### Brain Slice Preparation

Following rapid decapitation under isoflurane, brains were extracted and placed into ice-cold cutting solution containing (in mM): 194 sucrose, 30 NaCl, 4.5 KCl, 1 MgCl_2_, 26 NaHCO_3_, 1.2 NaH_2_PO_4_, 10 Glucose which was saturated with a mixture of 95% O_2_ and 5% CO_2_. Brains were sliced to a thickness of 280 µm on Leica VT1200S vibratome (Leica Microsystems) and slices were transferred to an artificial cerebrospinal fluid (aCSF) solution containing (in mM): 124 NaCl, 4.5 KCl, 1 MgCl_2_, 26 NaHCO_3_, 1.2 NaH_2_PO_4_, 10 Glucose, 2 CaCl_2_ (310–320 mOsm) saturated with 95% O_2_/5% CO_2_ at 36°C for 1 h before being moved to room temperature. Slices were transferred to a recording chamber continuously perfused with the aCSF solution saturated with 95% O_2_/5% CO_2_.

#### Electrophysiology Recordings

Whole-cell, voltage-clamp recordings from pyramidal neurons in layer 2/3 (L2/3) of the S1 barrel fields (between bregma −0.22 and −1.94 mm) were carried out at 29–32°C and aCSF was continuously perfused at a rate of 1–2 mL/min. Recordings were made from neurons using a Multiclamp 700B amplifier (Axon Instruments). Slices were visualized on an Olympus BX51WI microscope (Olympus Corporation of America). Pyramidal neurons were identified by their size, membrane resistance, and capacitance. Patch pipettes were prepared from filament-containing borosilicate micropipettes (World Precision Instruments) using a P-1000 micropipette puller (Sutter Instruments), having a 2.0–4.0 MΩ resistance. For both inhibitory and excitatory currents, tetrodotoxin (500 μM) was also added to the aCSF. For excitatory currents, the internal solution contained (in mM) 120 CsMeSO_3_, 5 NaCl, 10 TEA-Cl, 10 HEPES, 5 lidocaine bromide, 1.1 EGTA, 0.3 Na-GTP, and 4 Mg-ATP and picrotoxin (50 µM) was added to the aCSF for recordings to isolate excitatory transmission. For inhibitory currents, the internal solution contained (in mM): 120 CsCl_2_, 10 HEPES, 10 EGTA, 4 MgCl_2_, 2 MgATP, 0.5 NaGTP, and 5 lidocaine and 5 µM NBQX and 50 µM AP-5 were added to the aCSF for isolating inhibitory transmission.

After a stabilization period of at least 5 mins, miniature inhibitory postsynaptic currents or excitatory postsynaptic currents (mIPSCs and mEPSCs, respectively) were measured over the course of a 3-min gap-free recording for mEPSCs and 2 mins for mIPSCs. Data were acquired using Clampex 10.3 (Molecular Devices).

#### Electrophysiology Data Processing

For all recordings, series resistance was monitored and only cells with a stable series resistance (less than 25 MΩ and that did not change more than 15% during recording) were included for data analysis. mEPSC and mIPSC data were processed *via* MiniAnalysis software (Synaptosoft Inc.).

### Immunohistochemistry

Offspring were anesthetized with isoflurane and perfused with 4% paraformaldehyde prepared in PBS for 10 mins at a pump rate of ∼2 mL/min. Fixed brains were sectioned into 100 μm sections in the coronal plane (between bregma −0.1 and −1.94 mm) using a Leica VT-1000 vibrating microtome (Leica Microsystems) and stored in antigen preserved solution (PBS, 50% ethylene glycol and 1% polyvinyl pyrrolidone) at −20°C until later analysis. For synaptic marker (VGAT, Gephyrin, VGluT1, VGluT2, and PSD95) staining, sections were permeabilized with 2% Triton X100, then incubated with a blocking solution (3% normal goat serum prepared in PBS with 0.3% Triton X-100) and then incubated overnight with primary antibody prepared in blocking solution (See Table 1 for concentration and source). For S100β and Iba1 staining, sections were permeabilized with 0.3% Triton X100, then incubated with a blocking solution and then incubated overnight with primary antibody prepared in blocking solution. An appropriate secondary antibody conjugated with an Alexa series fluorophore was used to detect the primary antibody. DAPI (100 ng/ml, Thermo Fisher) or Draq5 (1:10,000 dilution, Cell Signaling) was included in the secondary antibody solution to stain nuclei.

**TABLE 1:** Antibody descriptions.

Antibodies	Host	Source	RRID#	Titer
Iba1/CD11b	Rabbit	WAKO chemicals (019-19741)	AB_839504	1:1000
S100β	Rabbit	Rockland antibodies and assays (600-401-379)	AB_882426	1:1000
VGAT	Rabbit	SYSY (131-002)	AB_887871	1:2000
Gephyrin	Mouse	SYSY(147-021)	AB_2232546	1:1000
PSD95	Rabbit	Thermo Fisher (51-6900)	AB_2533914	1:2000
VGluT1	Guinea Pig	Millipore (AB5905)	AB_2301751	1:2000
VGluT2	Guinea Pig	SYSY (135-404)	AB_887884	1:2000

For imaging synaptic marker staining, Z-stack confocal images were acquired from both hemispheres with a Nikon A1 confocal microscope with a 60X/NA1.4 objective at 3 times software zoom or Leica SP8 confocal microscope with a 63X/NA1.2 objective at 2.5 times software zoom. The Z-stacks were taken at 0.1 µm intervals (for VGAT + Gephyrin) or 0.2 µm (for VGluT1/2 + PSD95), and 2–4 µm-total thickness was imaged. Two images from each hemisphere and both hemispheres were imaged per animal. We utilized Imaris (Bitplane, Zurich, Switzerland) to quantify synaptic punctate at the three-dimensional level and establish the data analysis workflow to quantify the synaptic number according to the published literature (21–24). The volume occupied by nuclei and vasculature varied within each image, thus robustly impacting the density of synaptic marker quantification. To accurately estimate the neuropil occupied volume, we first used surface module to create the surface objects of nuclei and vasculature-like structure. Next, the gephyrin- or PSD95-channel was further masked by nuclei and vasculature objects to exclude the volume occupied by nuclei and vasculature. The post-masked gephyrin or PSD95 channel was used to generate a surface object containing the volume (neuropil object) to be analyzed. For spot detection, we followed similar procedures and parameter settings as described before (21–24). Specifically, the pre-synaptic (VGluT1, VGluT2, and VGAT) and postsynaptic (gephyrin and PSD95) punctate were detected by Imaris spot module with 0.5 µm and 0.3 µm diameter according to the published literature. In general, the diameter for synaptic puncta is between 0.25–0.8 µm (25, 26). In our experience in analyzing all acquired images (∼400 images), the automatic threshold by Imaris was unable to detect synaptic punctuates reliably. To find the optimal detecting threshold for spot detection, we first manually defined the detecting threshold for one image from each animal. The threshold that detected most synaptic punctates without creating artifacts was applied to analyze all images and generated the spot layer for each synaptic marker. Only synaptic punctates inside the neuropil-object were used for subsequence analysis. Next, we determine how many pre-synaptic spots were directly opposed to postsynaptic spots (defined as synapse at anatomical level) with the distance 0.5 µm. The juxtaposed synaptic punctate of VGluT1/PSD95, VGluT2/PSD95, and VGAT/gephyrin were defined as intracortical excitatory, thalamocortical excitatory, and inhibitory neurochemical inputs. Synaptic density was calculated as the number of synapses detected in a dataset over the volume of the dataset. All image acquisition and data analysis were performed in a blinded manner.

For visualizing S100β and Iba1 staining, Z-stack confocal images were acquired from both hemispheres with a Nikon A1 confocal microscope with a 10X/NA0.45 objective or Leica confocal microscope with a 10X/NA0.75 objective. The Z-stacks were taken at 1 µm intervals, 5 µm-total thickness was imaged. One image from each hemisphere and both hemispheres were imaged per animal. Projection images of 5 µm-thickness were used for image quantification by using NIH ImageJ software. If the location was damaged or folded and, thus, unable to be quantified, the image was discarded. All image acquisition and data analysis were performed in a blinded manner.

### Statistics and Data Exclusion

#### Gene Ontology Enrichment Analysis

All analyses are presented as PME relative to PSE (e.g., log2 abundance ratios of PME/PSE). For overrepresentation analysis of Gene Ontology (GO), the UniProt Accessions of all differentially abundant proteins (*p* < 0.05) were submitted to the g:Profiler g:GOst Functional Profiling platform (27). For settings, “only annotated genes” was selected for the statistical domain scope and the significance threshold was set to Benjamini-Hochberg FDR<0.05. Electronic GO annotations were excluded and the term size was filtered to between 5 and 2000. The full results of the GO analysis are provided in the Supplementary Material and at https://github.com/gggrecco/S1-Omics. The biological process (BP) and cellular component (CC) terms were exported and subsequently processed via REViGO (Reduce and Visualize Gene Ontology) to reduce redundancy, summarize, and better visualize GO enrichment as a network (28). This network was clustered using the AutoAnnotate plugin in CytoScape and further formatted to generate a publication-ready figure.

#### Kinase-Substrate Enrichment Analysis

A kinase-substrate enrichment analysis (KSEA) of the phosphoproteomics data was performed using the KSEA App (https://casecpb.shinyapps.io/ksea/) (29). All identified phosphopeptides with quantified abundance ratios (PME/PSE) and confirmed phosphosite modifications were utilized for the KSEA. PhosphoSitePlus + NetworKIN (NetworKIN score cutoff of 2) were used as the kinase-substrate dataset. Results were FDR-corrected (<0.05), and a z-score of enrichment was calculated to determine the normalized magnitude of upregulation or downregulation of kinases (PME vs. PSE). The full results of the KSEA analysis are provided in the Supplementary Material and at https://github.com/gggrecco/S1-Omics. The kinase scores resulting from the KSEA analysis were exported to Coral and overlayed onto kinome trees to better visualize patterns in kinase regulation where branches were set to represent the significance level, node color represents the z-score of enrichment, and node size represents the size of enrichment (absolute value of z-score) (30).

#### Electrophysiology and Immunohistochemistry Analysis

Data are graphically presented as the mean ± SEM for repeated measures or dot plots displaying all individual data points. The level of significance was *a priori* set at *p* < 0.05. All experiments were performed using both male and female offspring. To minimize potential litter effects in all completed studies, no more than two males and females per litter were utilized for any study. All studies were sufficiently powered to detect sex differences with sex considered as a factor. Immunostaining and electrophysiology statistical analyses were conducted using GraphPad Prism 9 software. ANOVAs with Sidak’s post hoc tests were used for analyzing all electrophysiology and immunostaining data.

## Results

### Multi-Omic Assessments of Prenatal Methadone Exposure

#### Differential Protein and Phosphopeptide Expression

**FIGURE 1:**
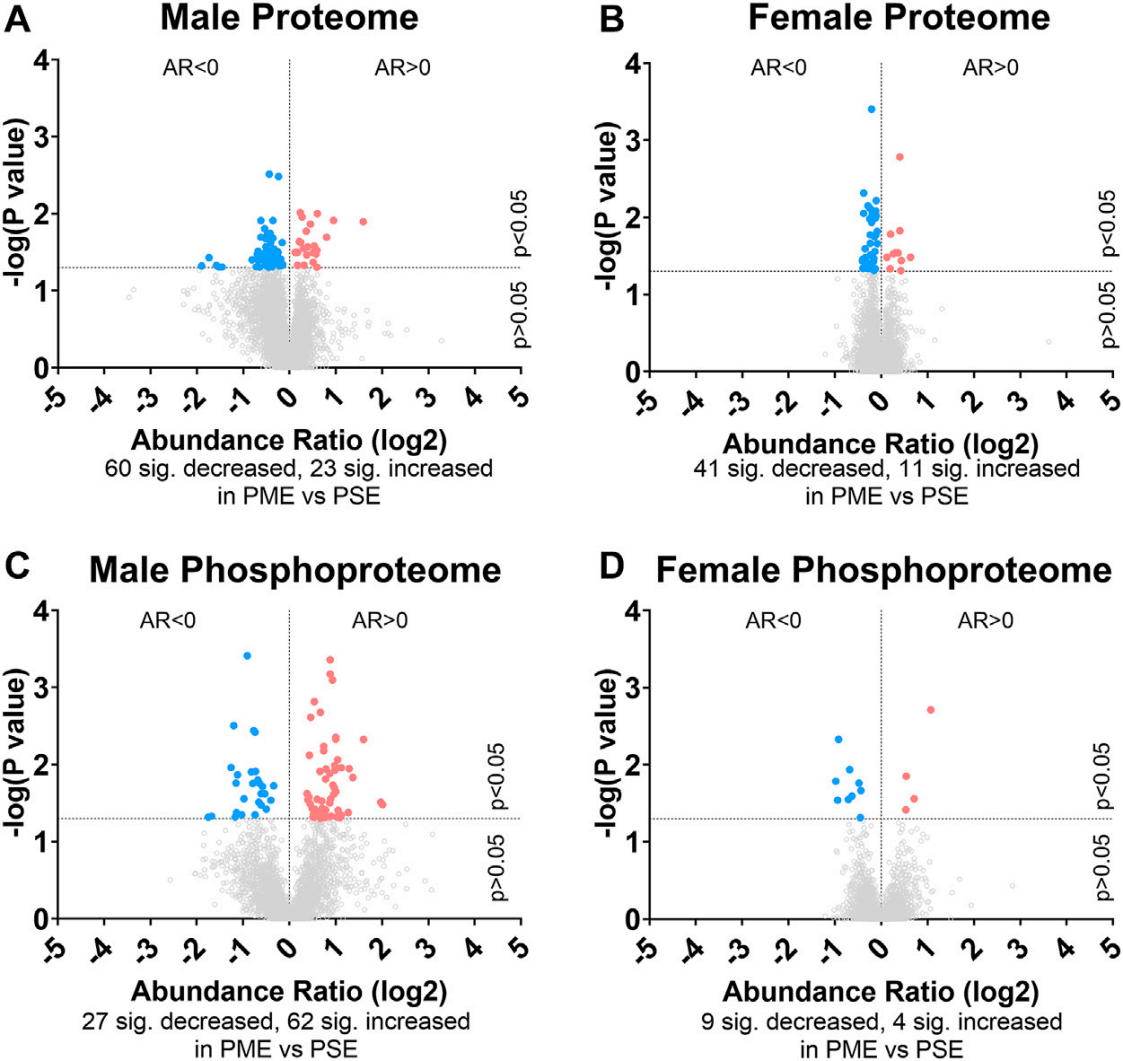
Differential protein and phosphopeptide expression in the somatosensory cortex of prenatal methadone exposed offspring. Volcano plots for the differential proteome in males **(A)** and females **(B)**, and phosphoproteome of males **(C)** and females **(D)** with blue circles representing individual proteins/phosphopeptides decreased in PME vs. PSE and red circles representing individual proteins/phosphopeptides increased in PME vs. PSE which reach the level of significance. AR, abundance ratio. *n* = 8 (4M:4F) PME, 8 PSE (4M:4F).

**FIGURE 2:**
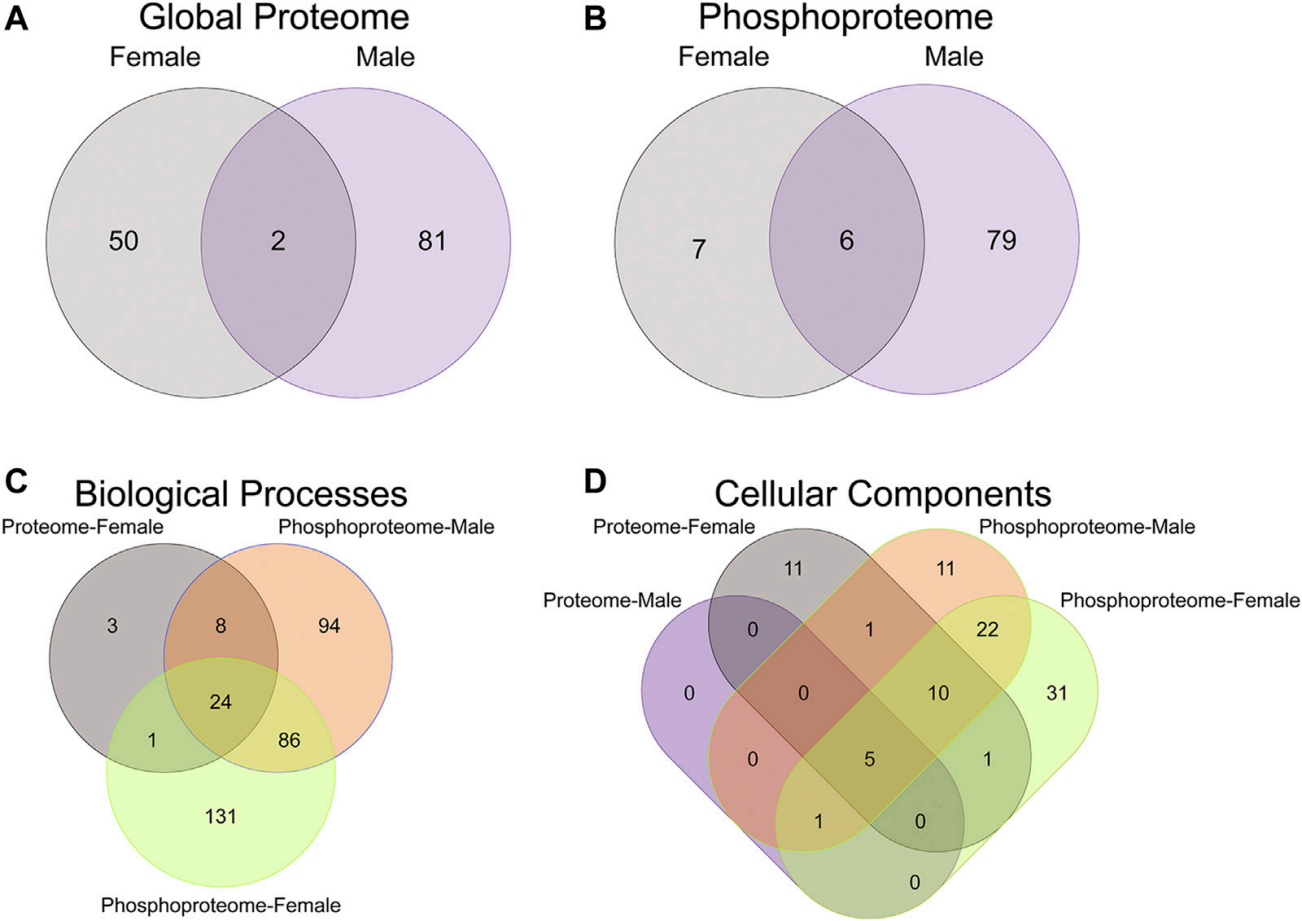
Overlap in the protein, phosphopeptides, and gene ontology enrichment between prenatal methadone exposed males and females. Of the significantly differentially abundant proteins in the global proteome **(A)** and phosphoproteome **(B)** of PME females (grey) and males (purple), only 2 proteins and 6 phosphorylated proteins were identified in both males and females. The overlap in enriched biological processes **(C)** and cellular components **(D)** revealed 24 biological processes terms and five cellular component terms that were identified in all completed gene ontology analyses.

To initiate an exploration into the possible mechanisms underlying the aberrant behavioral development in PME offspring, we collected whole S1 cortices from adolescent male and female PME and PSE offspring for quantitative proteomic and phosphoproteomic analysis. Overall, we identified 10,333 proteins and 3,231 phosphopeptides in the S1 of offspring. For the global proteome, 83 proteins were differentially abundant in males while 52 were differentially abundant in females with a majority of proteins showing reduced expression (*p* < 0.05; Figures 1A,B). For the phosphoproteome, 89 phosphopeptides were differentially abundant in males and 13 were differentially abundant in females (*p* < 0.05; Figures 1C,D). These differentially abundant proteins and phosphorylated proteins included synaptic vesicle release machinery (synaptotagmin, bassoon, VAT1, and RIMS1), ion channels (GluN2B, GlyR α4 subunit, and voltage-dependent L-type calcium channel β4), proteins associated with the postsynaptic signaling response (GRIP1, SAP90/PSD95-associated proteins, and CaMKIIβ), and various proteins associated with maintaining synaptic structure (microtubule associated proteins, ankyrin 3, and NCAM). For a full list of these proteins, abundances for each sample, and p values, please see the Supplementary File spreadsheet. Surprisingly, there was very little overlap in the differentially abundant proteins or phosphopeptides (Figures 2A,B) between PME males and PME females. These data suggest that PME has a sex-dependent impact on the S1 proteome and phosphoproteome.

#### Gene Ontology Functional Enrichment

**FIGURE 3:**
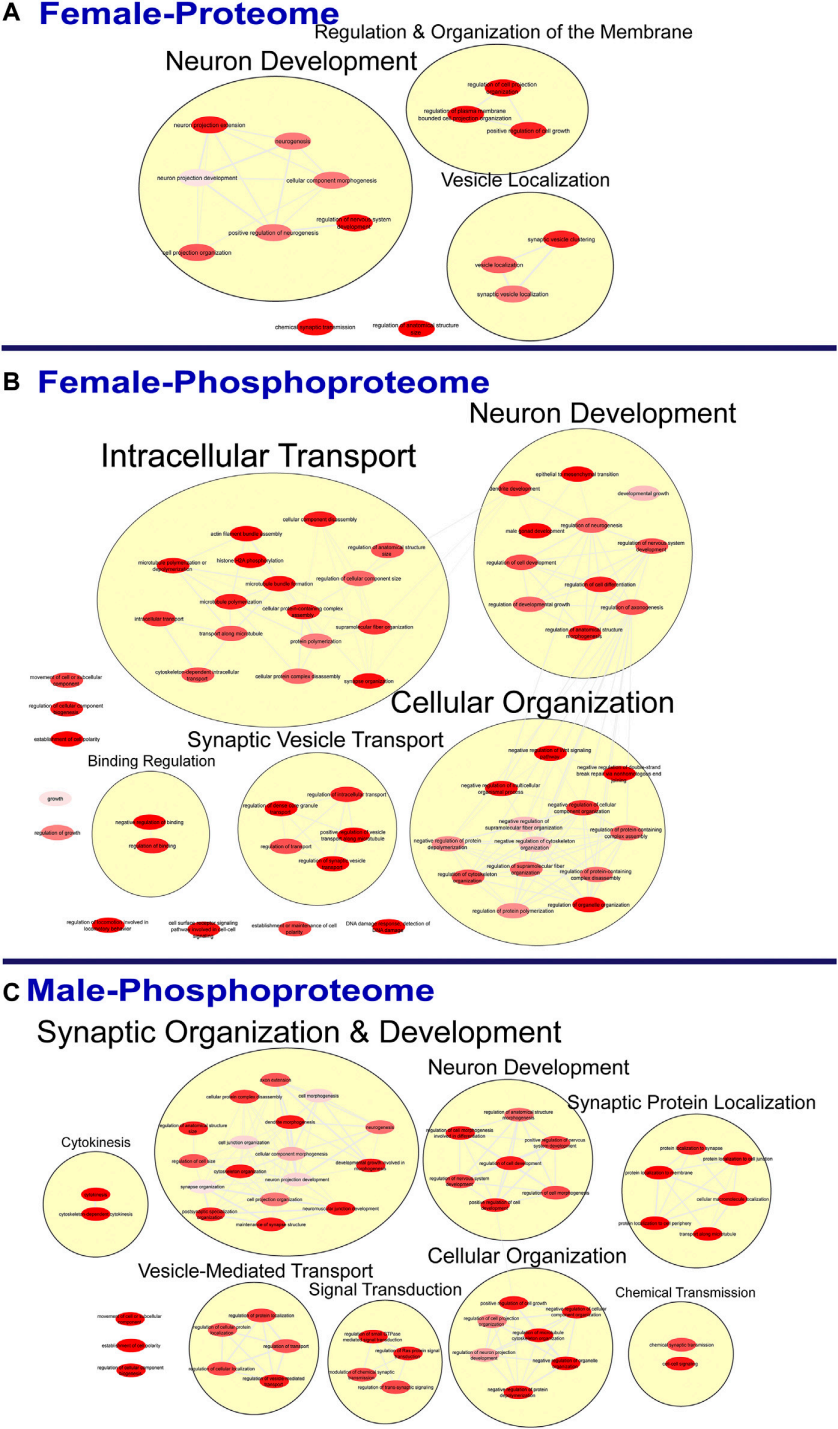
Clustering of enriched biological processes. Gene ontology enrichment analysis of biological processes enriched among the significant differentially abundant proteins in females **(A)** and differentially abundant phosphopeptides in females **(B)** and males **(C)** were reduced and clustered by REVIGO and visualized to facilitate identification of similarities. No enrichment was present in the males for the global proteome. The full results of the GO analysis for biological processes is provided in the Supplementary Material.

**FIGURE 4:**
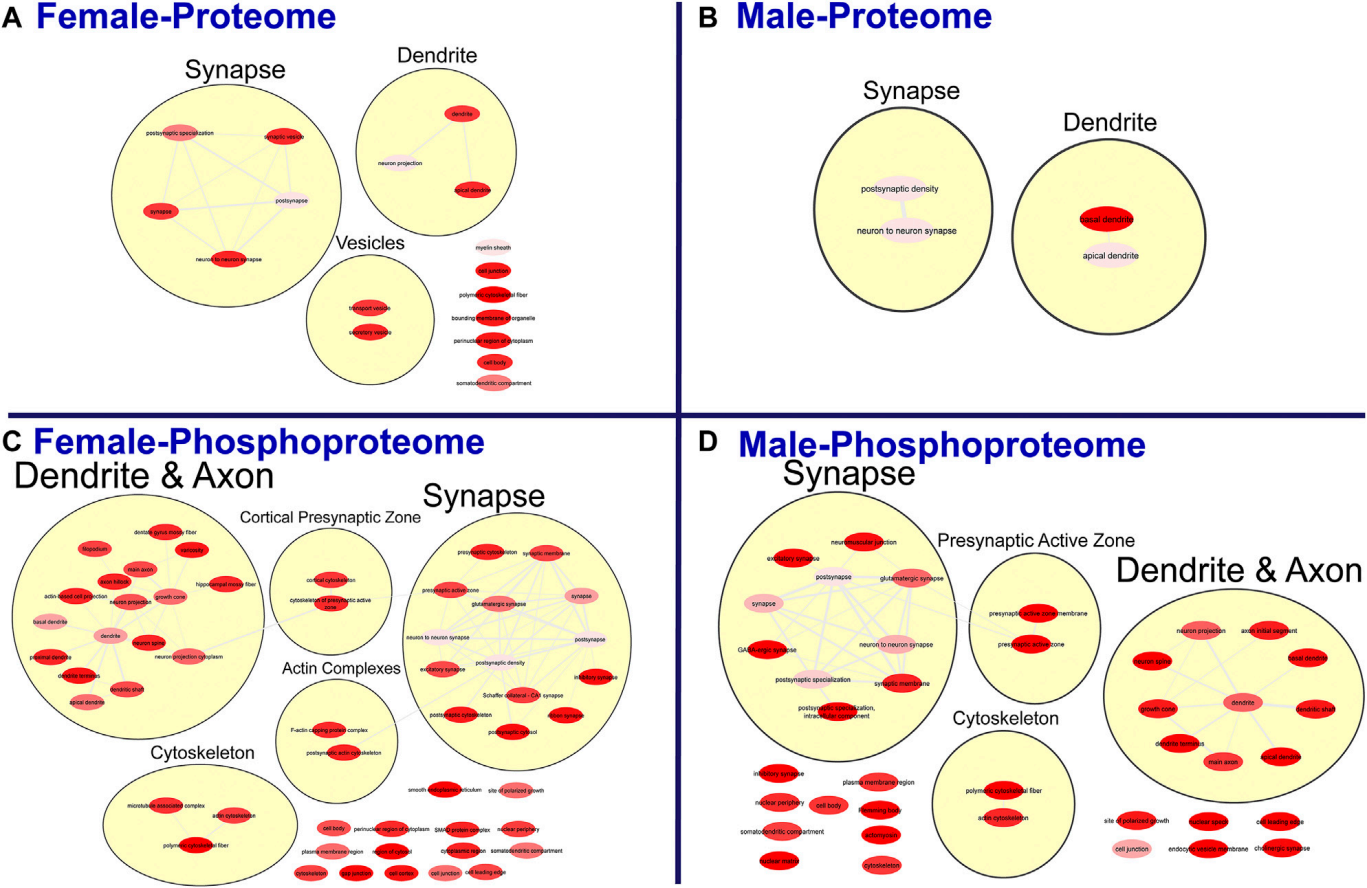
Clustering of enriched cellular components. Gene ontology enrichment analysis of cellular components enriched among the significant differentially abundant proteins in females **(A)** and males **(B)** and differentially abundant phosphopeptides in females **(C)** and males **(D)** were reduced and clustered by REVIGO and visualized to facilitate identification of similarities. The full results for the GO analysis for cellular components is provided in the Supplementary Material.

To further probe differences in the proteome and phosphoproteome of the S1, a Gene Ontology (GO) enrichment analysis to identify enriched Biological Processes (BPs) and Cellular Components (CCs) was performed using g:Profiler on the networks of significant differentially abundant proteins and phosphopeptides (27). The analysis of the global proteome revealed that 0 BPs and only 6 CCs in PME males while 36 BPs and 28 CCs in PME females were enriched in the network of differentially abundant proteins (FDR < 0.05). The phosphopeptide network appeared more enriched than the global proteome network. In the network of differentially abundant phosphopeptides of males and females, 212 BPs and 50 CCs in PME males and 242 BPs and 70 CCs in PME females were identified as enriched. The full identity and description of the terms can be found in the Supplementary Information. To facilitate the identification of patterns among the enriched terms, REViGO (28) was utilized to reduce redundancy and consolidate the similarities among the large lists of enriched BPs and CCs. In CytoScape, these terms were clustered into larger groups based on shared identities and visualized as nodes with edges indicating overlapping proteins associated with the BP or CC (Figures 3, 4, respectively). Many of the larger clusters of BPs enriched in the network of differentially abundant proteins and phosphorylated proteins were related to neuronal development, vesicle localization and transport, and synaptic organization (Figure 3). For the enriched CCs, large clusters were frequently associated with the synapse, dendrite, and axon of the neuron (Figure 4). Although there were few overlapping proteins/phosphopeptides identified as differentially abundant in both PME males and females, there were many more similarities in BPs and CCs that were enriched in the proteome and phosphoproteome of both PME males and females (Figures 2C,D). These network analyses suggest PME disrupts neuronal development and synaptic function through wide-scale changes in the proteomic and phosphoproteomic landscape.

#### Kinase-Substrate Enrichment Analysis

**FIGURE 5:**
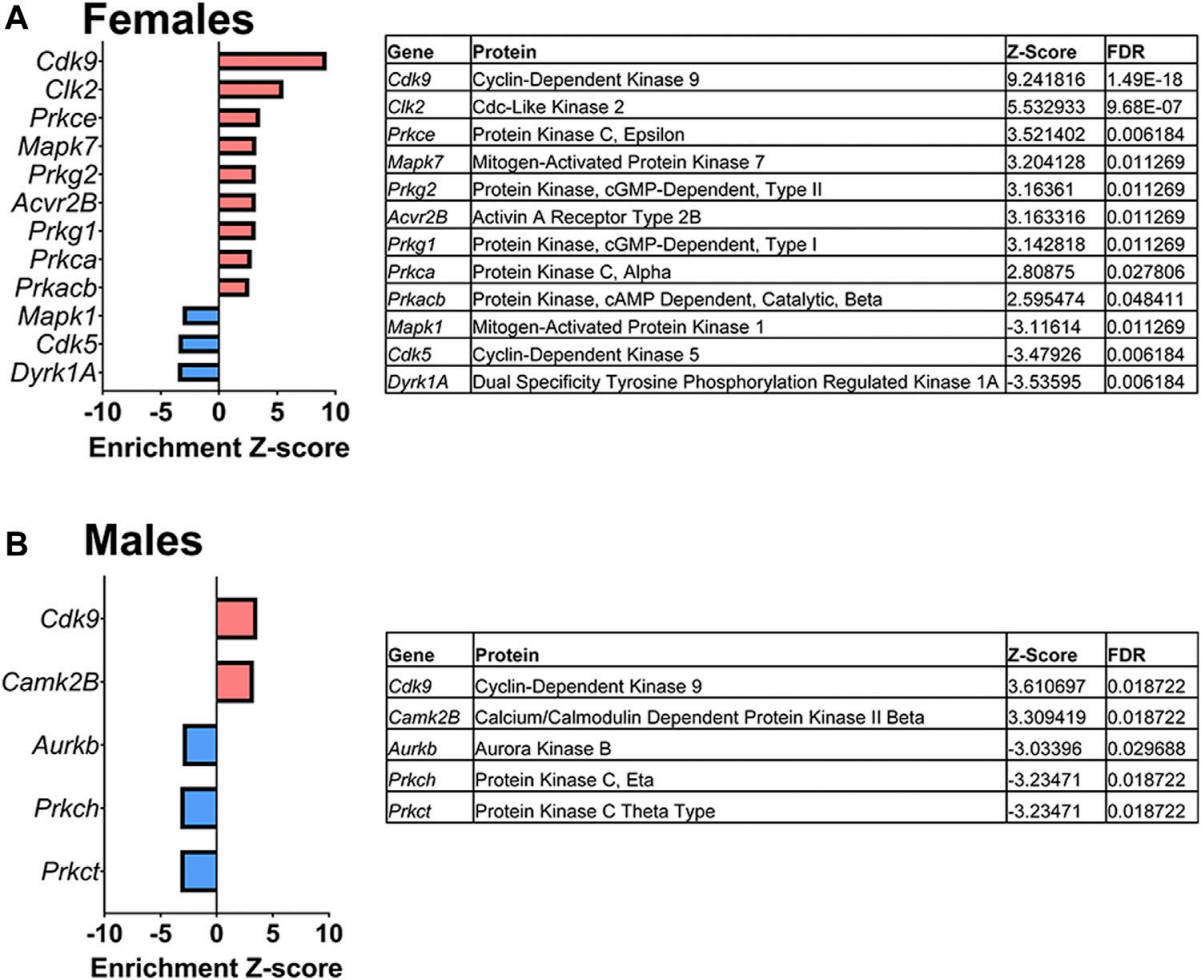
Dysregulated kinases. The results of a kinase-substrate enrichment analysis demonstrating the kinases that are significantly dysregulated (left; blue bars represent kinases predicted to be decreased in PME vs. PSE (FDR<0.05) and red bars represents kinases predicted to be increased in PME vs. PSE (FDR<0.05) with gene identities, protein names, z-score of enrichment, and FDR significance values provided in the tables (right) for females **(A)** and males **(B)**.

**FIGURE 6:**
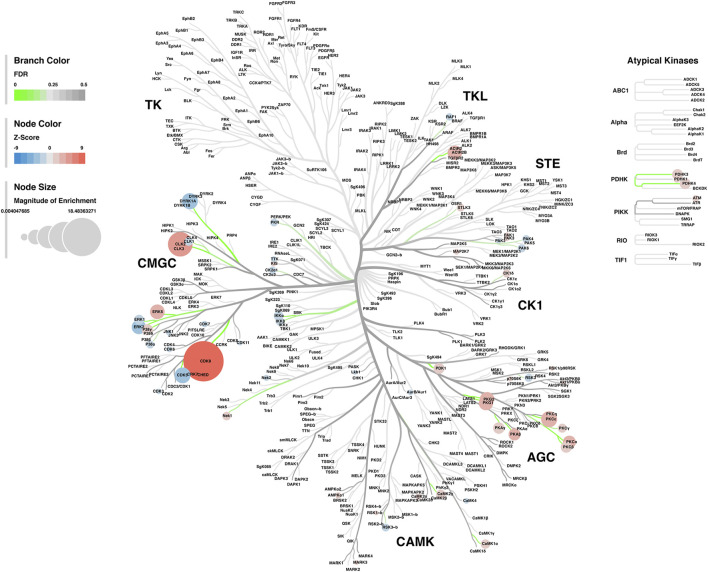
Kinome tree plot in females. The results from kinase-substrate enrichment analysis were mapped onto kinome treeplots *via* Coral in which branch color corresponds to significance level, node color corresponds to z-score of enrichment, and node size correspond to magnitude of enrichment for kinase pathways in the S1 of females.

**FIGURE 7:**
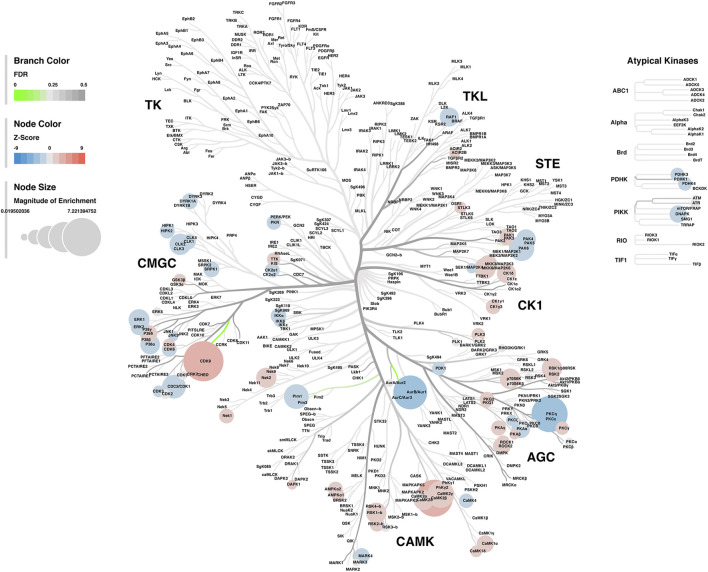
Kinome tree plot in males. The results from kinase-substrate enrichment analysis were mapped onto kinome treeplots *via* Coral in which branch color corresponds to significance level, node color corresponds to z-score of enrichment, and node size correspond to magnitude of enrichment for kinase pathways in the S1 of males.

Lastly, a kinase-substrate enrichment analysis (KSEA) was performed (29) to estimate the changes in kinase pathways based on specific phosphorylation site modifications. The significant kinases predicted to be disrupted in the S1 of PME males and females can be seen in Figures 5A,B, respectively. The full enrichment results of the KSEA kinase scores and significance threshold is found in the Supplementary Material. The KSEA output was then overlaid onto kinome trees using Coral (30) to visualize patterns in enrichment among the various kinase families (Figures 6, 7). In both females (Figure 6) and males (Figure 7), prenatal exposure to methadone was associated with many changes in the CMGC kinases (cyclin-dependent, mitogen-activated, glycogen synthase and CDC-like kinases family) with notable differences in the cyclin-dependent kinases (Cdk9, Cdk5, Cdk1, and Cdk6). In postmitotic neurons, it is generally thought that most Cdks display low expression; however, Cdk5 has been shown to phosphorylate presynaptic and postsynaptic proteins in mature neurons suggesting Cdks may impact plasticity and neurotransmission in postmitotic neurons (31, 32). The AGC kinases (protein kinase A, G, and C family) including PKC, PKA, and PKG and the CAMK kinases (Calcium and Calmodulin-regulated kinase family) including CaMKII, CaMK1, and CaMK4 were also predicted to be disrupted based on the differential phosphopeptide expression data. Members of the AGC and CAMK kinases families are well-known kinases regulating second messenger signaling cascades involved in synaptic signaling.

In summary, these multi-omic data indicate PME induces persistent and widespread changes to the S1 proteome and phosphoproteome with many effects associated with processes related to neurotransmission at the synapse in a sex-dependent manner.

### Inhibitory Synapses

#### Neurochemical Assessment of GABAergic Synaptic Markers

**FIGURE 8:**
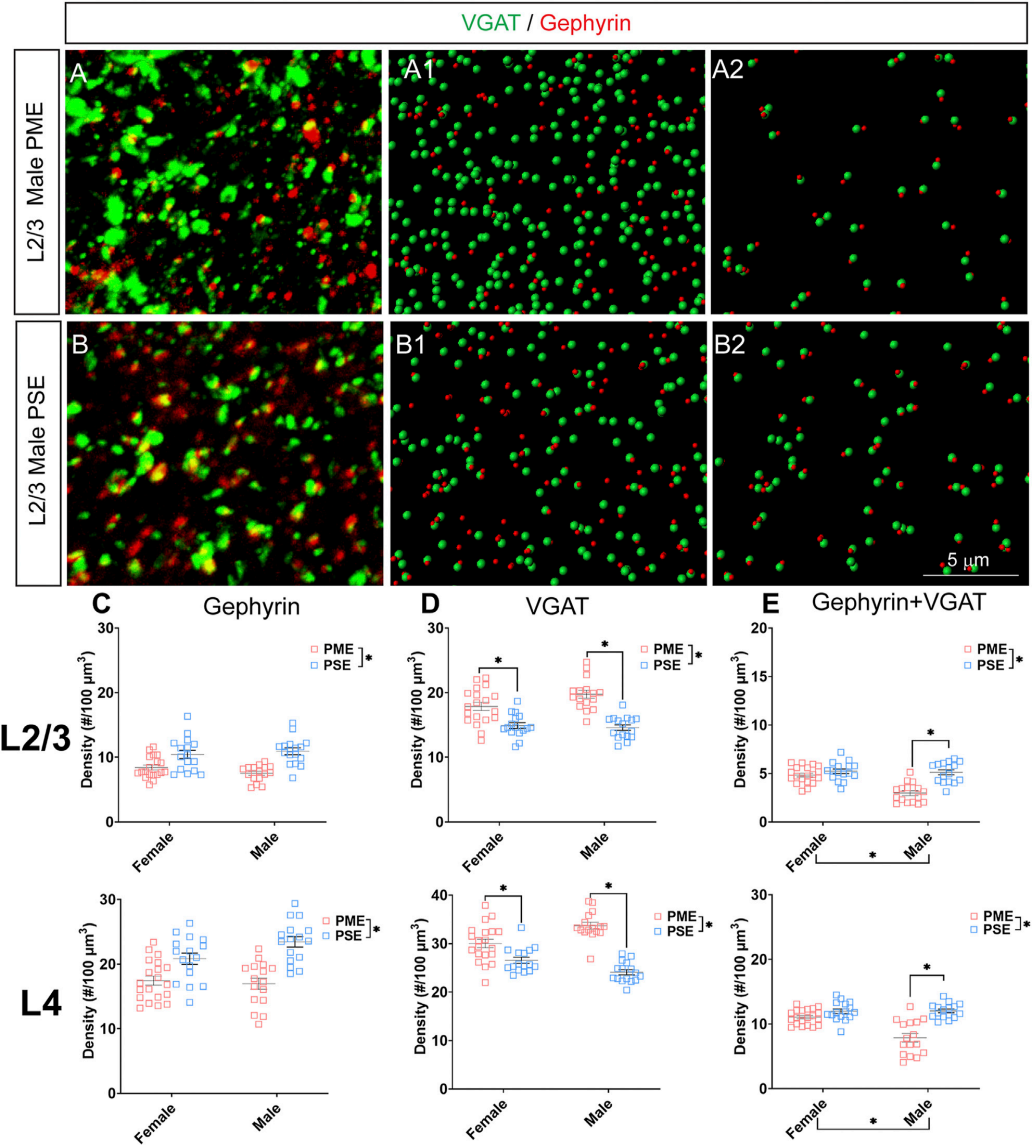
Neurochemical assessment of functional GABAergic synapses. In a PME male **(A)** and a PSE male **(B)** in L2/3, an exemplary confocal stack of post-synaptic density (gephyrin) and vesicular GABA transporter (VGAT) double-stained image in L2/3. The use of the spot detection in Imaris to identify the gephyrin+ and VGAT + puncta **(A1,B1)**. The gephyrin and VGAT spot pairs in the vicinity of 0.5 µm, which defined neurochemical VGAT input synapses **(A2,B2)**. **(C)** PME significantly reduces gephyrin densities in L2/3 (ANOVA: Exposure, *p* < 0.0001; top) and in L4 (ANOVA: Exposure, *p* < 0.0001; bottom). **(D)** PME significantly increases VGAT densities in L2/3 (ANOVA: Interaction, *p* = 0.049; PME female vs. PSE female, *p* = 0.0005; PME male vs. PSE male, *p* < 0.0001; top) and in L4 (ANOVA: Interaction, *p* < 0.0001; PME female vs. PSE female, *p* = 0.0017; PME male vs. PSE male, *p* < 0.0001; bottom). **(E)** PME significantly reduced functional GABAergic synapses in L2/3 (ANOVA: Interaction, *p* = 0.0004; PME male vs. PSE male, *p* < 0.0001; top) and L4 (ANOVA: Interaction, *p* = 0.0002; PME male vs. PSE male, *p* < 0.0001; top). *n* = 9 (4M:5F) PME, 8 PSE (4M:4F). Two images per hemisphere, both hemispheres were quantified in each animal. *p < 0.05.

Given the large number of differentially abundant proteins associated with synaptic functioning and the enrichment in terms associated with the synapse, we investigated GABAergic synapse density in layer 2/3 (L2/3) and layer 4 (L4) of the S1 using the co-localization of the presynaptic vesicular protein VGAT and postsynaptic protein gephyrin (See Figures 8A,B for representative images). The co-localization VGAT and gephyrin was used to infer the presence of a “functional” GABAergic synapse from this anatomical data. Density of gephyrin was significantly reduced in both sexes as a result of PME in both L2/3 (ANOVA: Exposure, F_(1,63)_ = 32.39, *p* < 0.0001; Sex, F_(1,63)_ = 0.161, *p* = 0.69; Interaction, F_(1,63)_ = 2.12, *p* = 0.15; Figure 8C, top) and L4 (ANOVA: Exposure, F_(1,64)_ = 37.30, *p* < 0.0001; Sex, F_(1,64)_ = 1.76, *p* = 0.19; Interaction, F_(1,64)_ = 3.64, *p* = 0.061; Figure 8C, bottom). For VGAT, PME significantly increased density in both L2/3 (ANOVA: Exposure, F_(1,63)_ = 54.29, *p* < 0.0001; Sex, F_(1,63)_ = 2.02, *p* = 0.16; Interaction, F_(1,63)_ = 3.32, *p* = 0.049; PME female vs. PSE female, *p* = 0.0005; PME male vs. PSE male, *p* < 0.0001; Figure 8D, top) and L4 (ANOVA: Exposure, F_(1,64)_ = 81.24, *p* < 0.0001; Sex, F_(1,64)_ = 0.675, *p* = 0.41; Interaction, F_(1,64)_ = 17.70, *p* < 0.0001; PME female vs. PSE female, *p* = 0.0017; PME male vs. PSE male, *p* < 0.0001; Figure 8D, bottom). Although a main effect of exposure was present on the co-localization of gephyrin and VGAT in both L2/3 and L4, this exposure effect appeared to be driven by PME males which exhibited a significantly reduced density of co-localization in both L2/3 (ANOVA: Exposure, F_(1,63)_ = 29.14, *p* < 0.0001; Sex, F_(1,63)_ = 17.75, *p* < 0.0001; Interaction, F_(1,63)_ = 13.93, *p* = 0.0004; PME female vs. PSE female, *p* = 0.41; PME male vs. PSE male, *p* < 0.0001; Figure 8E, top) and L4 (ANOVA: Exposure, F_(1,64)_ = 38.68, *p* < 0.0001; Sex, F_(1,64)_ = 14.98, *p* = 0.0003; Interaction, F_(1,64)_ = 16.06, *p* = 0.0002; PME female vs. PSE female, *p* = 0.21; PME male vs. PSE male, *p* < 0.0001; Figure 8E, bottom). These findings indicate PME significantly reshapes GABAergic synapse development in PME offspring by reducing the number of putative functional GABAergic synapses, although this effect is more prominent in male offspring.

#### Electrophysiological Assessment of Inhibitory Neurotransmission

**FIGURE 9:**
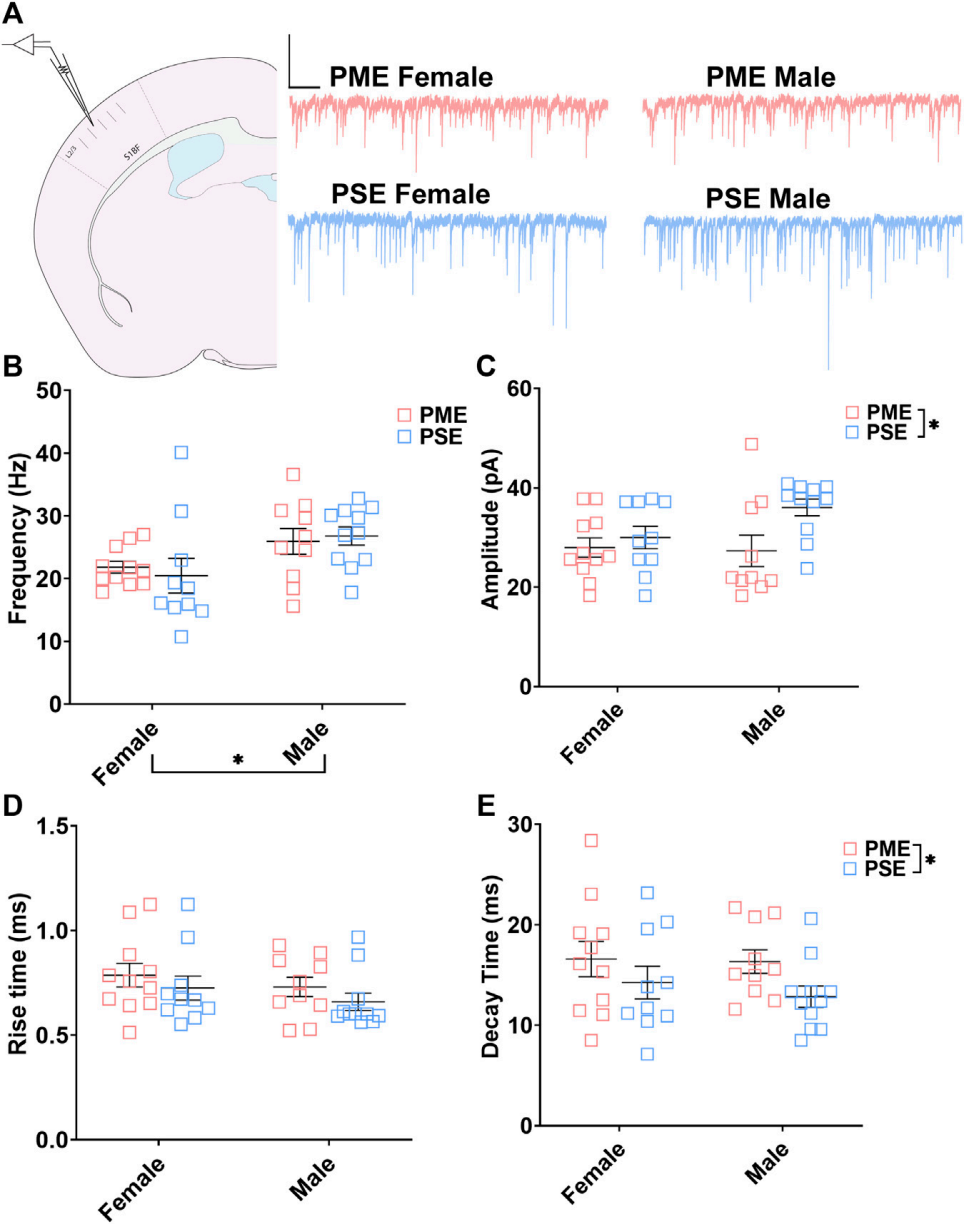
PME impairs inhibitory transmission in L2/3 pyramidal neurons. **(A)** Schematic demonstrating coronally sectioned brain slice to acquire the S1 barrel fields (S1BF) for whole cell voltage clamp recordings at approximately −0.82 mm bregma (*left*). Representative traces for miniature inhibitory postsynaptic currents (mIPSCs) in the S1 (*right*). Scale Bars = 500 ms, 100 mV **(B)** mIPSC frequency was not affected by PME. **(C)** The amplitude of mIPSCs was significantly reduced in PME offspring (ANOVA: Exposure, *p* = 0.024). **(D)** The rise time was not altered by prenatal exposure. **(E)** The decay constant was significantly lengthened in PME offspring (ANOVA: Exposure, *p* = 0.049). *n* = 8 PME mice (4M:4F), 21 neurons (10M:11F) and 8 PSE mice (4M:4F), 21 neurons (11M:10F). *p < 0.05.

These neurochemical differences in GABAergic synaptic markers led us to functionally examine inhibitory neurotransmission (primarily GABAergic) at L2/3 pyramidal neurons using whole cell patch clamp electrophysiology. Representative traces for mIPSCs in the S1 can be found in Figure 9A. The frequency of mIPSCs was significantly affected by sex but not prenatal exposure (ANOVA: Exposure, F_(1,38)_ = 0.017, *p* = 0.90; Sex, F_(1,38)_ = 7.77, *p* = 0.0083; Interaction, F_(1,38)_ = 0.349, *p* = 0.56; Figure 9B). PME offspring exhibited a significant decrease in the amplitude of mIPSCs compared to PSE offspring (ANOVA: Exposure, F_(1,38)_ = 5.51, *p* = 0.024; Sex, F_(1,38)_ = 1.37, *p* = 0.25; Interaction, F_(1,38)_ = 2.16, *p* = 0.15; Figure 9C), and similar to the co-localization analyses in the prior section, this effect on amplitude appears to be driven by PME males. No exposure-related effects were discovered for mIPSC rise time (ANOVA: Exposure, F_(1,38)_ = 1.71, *p* = 0.20; Sex, F_(1,38)_ = 1.46, *p* = 0.23; Interaction, F_(1,38)_ = 0.009, *p* = 0.92; Figure 9D). However, PME significantly lengthened the decay constant (ANOVA: Exposure, F_(1,38)_ = 4.14, *p* = 0.049; Sex, F_(1,38)_ = 0.333, *p* = 0.57; Interaction, F_(1,38)_ = 0.164, *p* = 0.69; Figure 9E). These electrophysiological findings indicate the differences in neurochemical synaptic markers may have functional consequences for inhibitory transmission in L2/3 pyramidal neurons of the S1.

### Excitatory Synapses

#### Neurochemical Assessment of Glutamatergic Synaptic Markers

**FIGURE 10:**
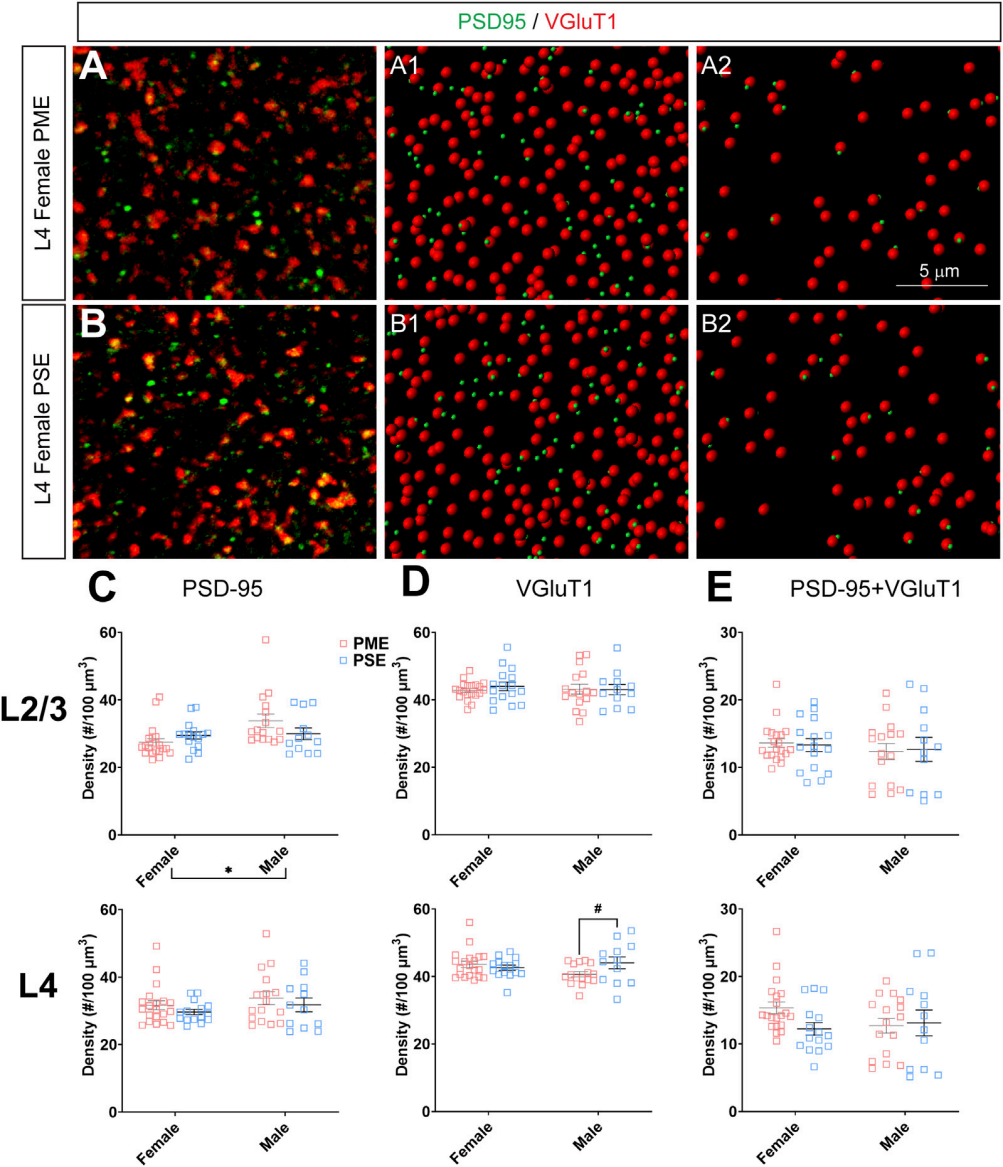
Neurochemical assessment of functional intracortical glutamatergic synapses. In a PME female **(A)** and a PSE female **(B)** in L4, an exemplary confocal stack of post-synaptic density (PSD95) and vesicular glutamatergic transporter 1 (VGluT1) double-stained image in L4. The use of the spot detection in Imaris to identify the PSD95+ and VGluT1+ puncta **(A1,B1)**. The PSD95 and VGluT1 spot pairs in the vicinity of 0.5 µm, which defined neurochemical VGluT1 input synapses **(A2,B2)**. **(C)** Although a main effect of sex was present, there was not an effect of exposure on PSD-95 density in L2/3 (top) or L4 (bottom). **(D)** There was no change in VGluT1 density in L2/3 (top), although there was a trend for decreased VGluT1 density in PME males (ANOVA: Interaction, *p* = 0.043; *p* = 0.069; bottom). **(E)** PME did not impact PSD-95 and VGluT1 co-localization in L2/3 (*top*) or L4 (bottom). *n* = 9 (4M:5F) PME, 8 PSE (4M:4F). Two image per hemisphere per animal. *p < 0.05. #*p* = 0.069.

**FIGURE 11:**
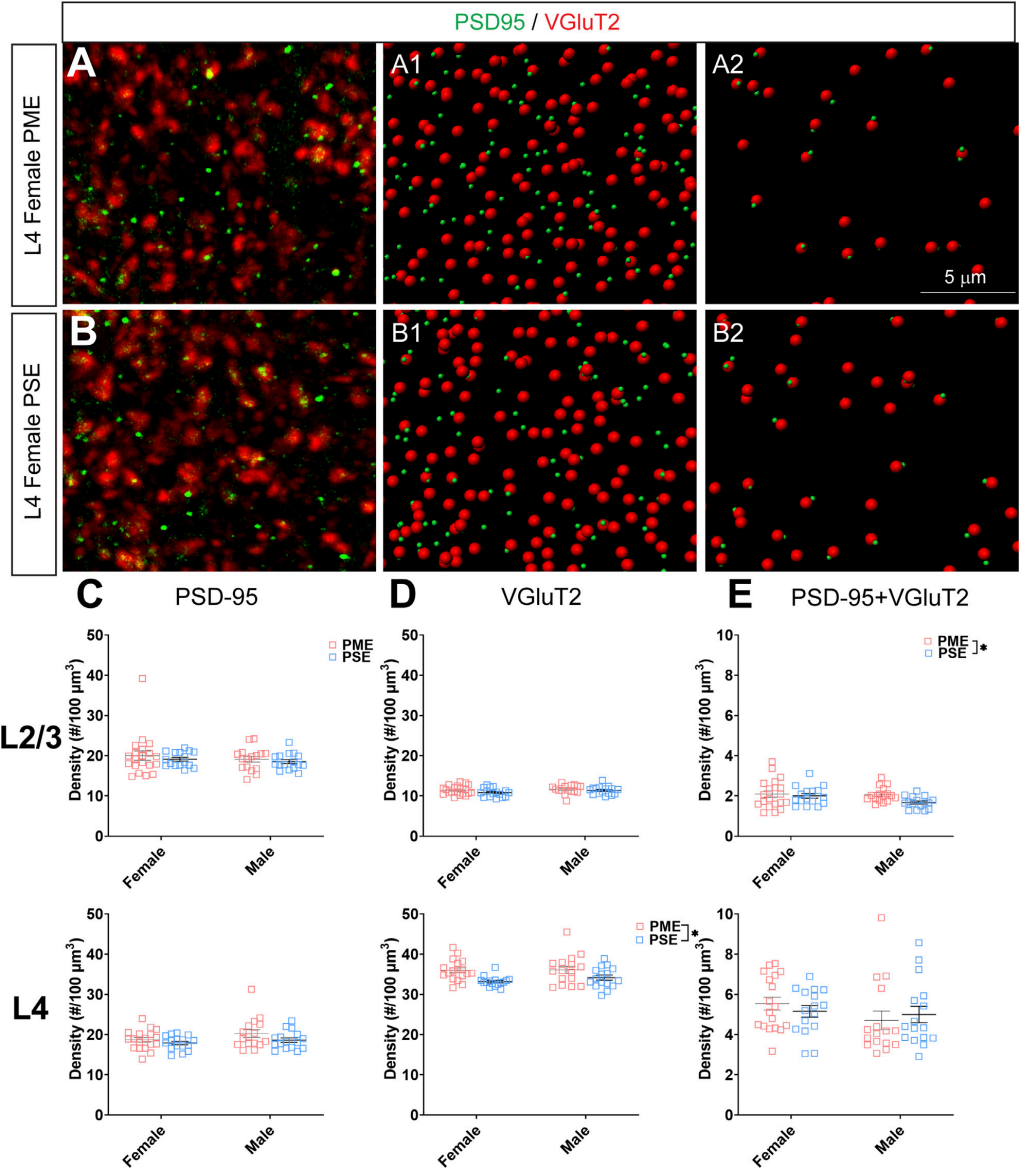
Neurochemical assessment of functional thalamocortical glutamatergic synapses. In a PME female **(A)** and a PSE female **(B)** in L4, an exemplary confocal stack of post-synaptic density (PSD95) and vesicular glutamatergic transporter 2 (VGluT2) double-stained image in L4. The use of the spot detection in Imaris to identify the PSD95+ and VGluT2+ puncta **(A1,B1)**. The PSD95 and VGluT2 spot pairs in the vicinity of 0.5 µm, which defined neurochemical VGluT2 input synapses **(A2,B2)**. **(C)** No exposure-related effects on PSD-95 density were present in L2/3 (top) or L4 (bottom). **(D)** There was no change in VGluT2 density in L2/3 (top); however, VGluT2 density was significantly increased in PME offspring in L4 (ANOVA: Exposure, *p* = 0.0005; bottom). **(E)** PME significantly increased PSD-95 and VGluT2 co-localization in L2/3 (ANOVA: Exposure, *p* = 0.049; top) but not in L4 (bottom). *n* = 9 (4M:5F) PME, 8 PSE (4M:4F). Two image per hemisphere per animal. *p < 0.05.

To investigate if the impairments in inhibitory synapses in PME offspring also extended to excitatory synapses, we next assessed glutamatergic synapse density in L2/3 and L4 of the S1 using the co-localization of the presynaptic vesicular protein VGluT1 (conventionally considered a marker of intracortical inputs; See Figures 10A,B for representative images) or VGluT2 (conventionally considered a marker thalamocortical inputs; See Figures 11A,B for representative images) and the postsynaptic protein PSD-95. The co-localization VGluT1 or VGluT2 with PSD-95 was used to infer the presence of a “functional” glutamatergic synapse from this anatomical data. Although there were numerous exposure-related effects on GABAergic synapses, disruptions in glutamatergic synaptic markers were less prominent. Density of PSD-95 was not affected by PME in either L2/3 (ANOVA: Exposure, F_(1,60)_ = 0.374, *p* = 0.54; Sex, F_(1,60)_ = 5.37, *p* = 0.024; Interaction, F_(1,60)_ = 3.94, *p* = 0.052; Figure 10C, top) or L4 (ANOVA: Exposure, F_(1,59)_ = 1.61, *p* = 0.21; Sex, F_(1,59)_ = 1.78, *p* = 0.19; Interaction, F_(1,59)_ = 0.000, *p* = 0.99; Figure 10C, bottom). PME also did not significantly affect density of VGluT1 in L2/3 (ANOVA: Exposure, F_(1,60)_ = 0.208, *p* = 0.65; Sex, F_(1,60)_ = 0.0533, *p* = 0.82; Interaction, F_(1,60)_ = 0.322, *p* = 0.57; Figure 10D, top). A significant interaction was present in L4 for VGluT1 density (ANOVA: Exposure, F_(1,59)_ = 1.30, *p* = 0.26; Sex, F_(1,59)_ = 0.483, *p* = 0.49; Interaction, F_(1,59)_ = 4.28, *p* = 0.043; Figure 10D, bottom), but post-hoc tests did not quite reach the level of significance (PME females vs. PSE females, *p* = 0.069). Co-localization of PSD-95 and VGluT1 was also not impacted by PME in either L2/3 (ANOVA: Exposure, F_(1,60)_ = 0.000, *p* = 0.99; Sex, F_(1,60)_ = 0.766, *p* = 0.39; Interaction, F_(1,60)_ = 0.079, *p* = 0.78; Figure 10E, top) or L4 (ANOVA: Exposure, F_(1,59)_ = 1.33, *p* = 0.25; Sex, F_(1,59)_ = 0.565, *p* = 0.46; Interaction, F_(1,59)_ = 2.27, *p* = 014; Figure 10E, bottom) suggesting intracortical glutamatergic synapses are not significantly disrupted by PME.

When assessing VGluT2 co-localization with PSD-95, we also did not discover any exposure-related effects on PSD-95 density in L2/3 (ANOVA: Exposure, F_(1,64)_ = 0.805, *p* = 0.37; Sex, F_(1,64)_ = 0.827, *p* = 0.37; Interaction, F_(1,64)_ = 0.0483, *p* = 0.83; Figure 11C, top) or L4 (ANOVA: Exposure, F_(1,62)_ = 3.35, *p* = 0.072; Sex, F_(1,62)_ = 2.92, *p* = 0.093; Interaction, F_(1,62)_ = 0.417, *p* = 0.52; Figure 11C, bottom). Although the increase in density of VGluT2 density in L2/3 did not reach the level of significance (ANOVA: Exposure, F_(1,64)_ = 2.71, *p* = 0.10; Sex, F_(1,64)_ = 1.94, *p* = 0.17; Interaction, F_(1,64)_ = 0.370, *p* = 0.54; Figure 11D, top), PME significantly increased VGluT2 density in L4 (ANOVA: Exposure, F_(1,62)_ = 13.37, *p* = 0.0005; Sex, F_(1,62)_ = 0.664, *p* = 0.42; Interaction, F_(1,62)_ = 0.484, *p* = 0.49; Figure 11D, bottom). PME increased co-localization of PSD-95 and VGluT2 in L2/3 (ANOVA: Exposure, F_(1,64)_ = 3.99, *p* = 0.049; Sex, F_(1,64)_ = 2.11, *p* = 0.15; Interaction, F_(1,64)_ = 1.56, *p* = 0.22; Figure 11E, top) but not in L4 (ANOVA: Exposure, F_(1,62)_ = 0.016, *p* = 0.90; Sex, F_(1,62)_ = 1.76, *p* = 0.19; Interaction, F_(1,62)_ = 0.805, *p* = 0.37; Figure 11E, bottom). This neurochemical assessment of putative functional glutamatergic synapses indicate PME may increase thalamocortical inputs in L4 and increase thalamocortical synaptic connections in L2/3.

#### Electrophysiological Assessment of Excitatory Neurotransmission

**FIGURE 12:**
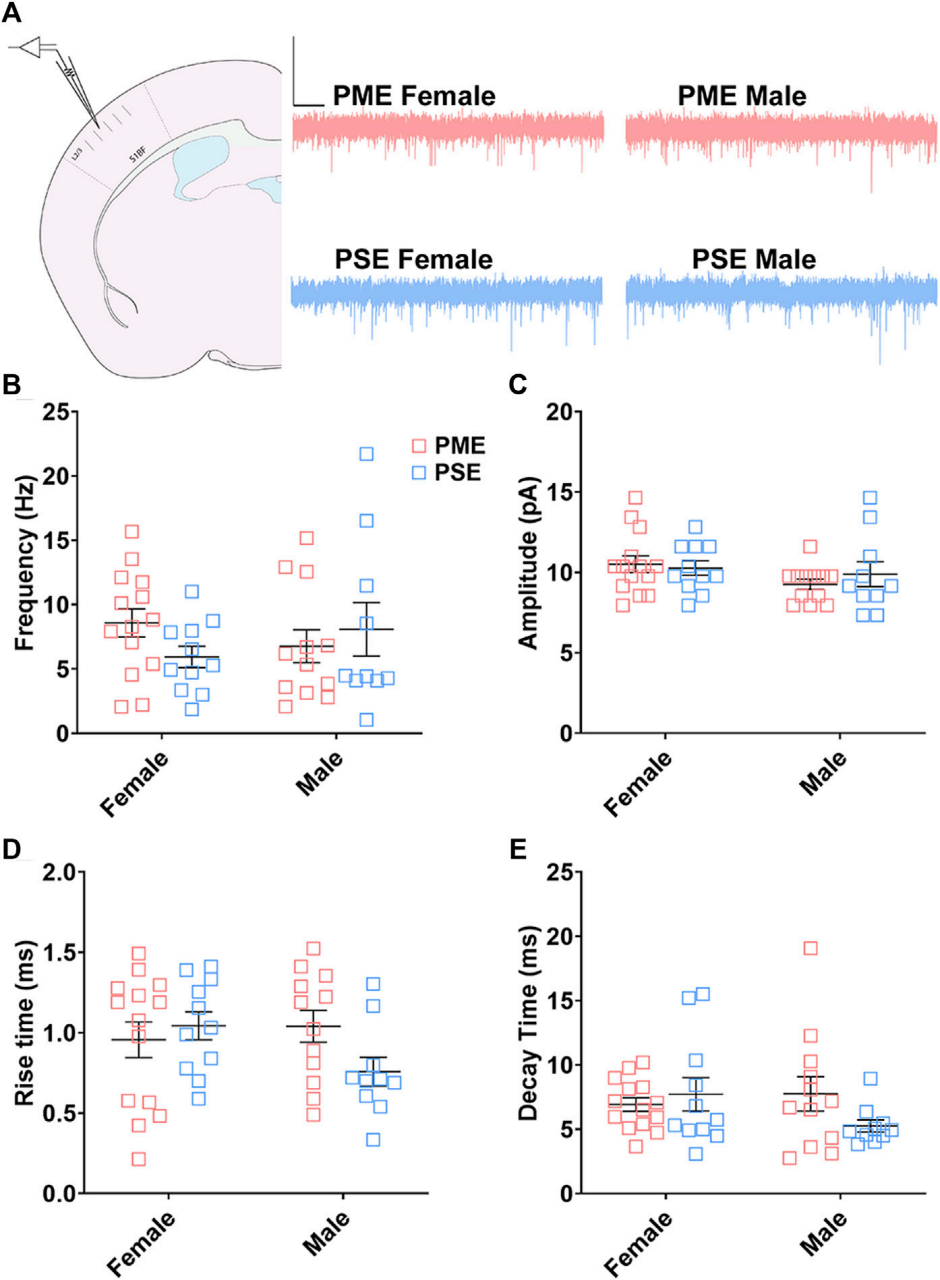
Excitatory transmission is not altered in L2/3 Pyramidal Neurons. **(A)** Schematic demonstrating coronally sectioned brain slice to acquire the S1 barrel fields (S1BF) for whole cell voltage clamp recordings at approximately −0.82 mm bregma (*left*). Representative traces for miniature excitatory postsynaptic currents (mEPSCs) in the S1 (*right*). Scale Bars = 500 ms, 50 mV. PME did not impact mEPSC **(B)** frequency, **(C)** amplitude, **(D)** rise time, or **(E)** decay constant. *n* = 8 PME mice (4M:4F), 26 neurons (12M:14F) and 8 PSE mice (4M:4F), 22 neurons (12M:10F).

We followed-up this neurochemical assessment of glutamatergic synaptic markers by assessing functional excitatory inputs to L2/3 pyramidal neurons using electrophysiology. Representative traces for mEPSCs in the S1 can be found in Figure 12A. There were no effects of sex or exposure on mEPSC frequency (ANOVA: Exposure, F_(1,43)_ = 0.248, *p* = 0.62; Sex, F_(1,43)_ = 0.014, *p* = 0.90; Interaction, F_(1,43)_ = 2.16, *p* = 0.15; Figure 12B), amplitude of responses (ANOVA: Exposure, F_(1,43)_ = 0.136, *p* = 0.71; Sex, F_(1,43)_ = 2.38, *p* = 0.13; Interaction, F_(1,43)_ = 0.684, *p* = 0.41; Figure 12C), rise times (ANOVA: Exposure, F_(1,43)_ = 0.942, *p* = 0.34; Sex, F_(1,43)_ = 0.991, *p* = 0.33; Interaction, F_(1,43)_ = 3.331, *p* = 0.07; Figure 12D), or decay constants (ANOVA: Exposure, F_(1,43)_ = 0.744, *p* = 0.39; Sex, F_(1,43)_ = 0.680, *p* = 0.41; Interaction, F_(1,43)_ = 2.77, *p* = 0.10; Figure 12E). Similar to the limited effects of PME on neuroanatomical data, PME does not appear to disrupt excitatory transmission in L2/3 pyramidal neurons of the S1.

### Glial Cell Density and Cortical Thickness

**FIGURE 13:**
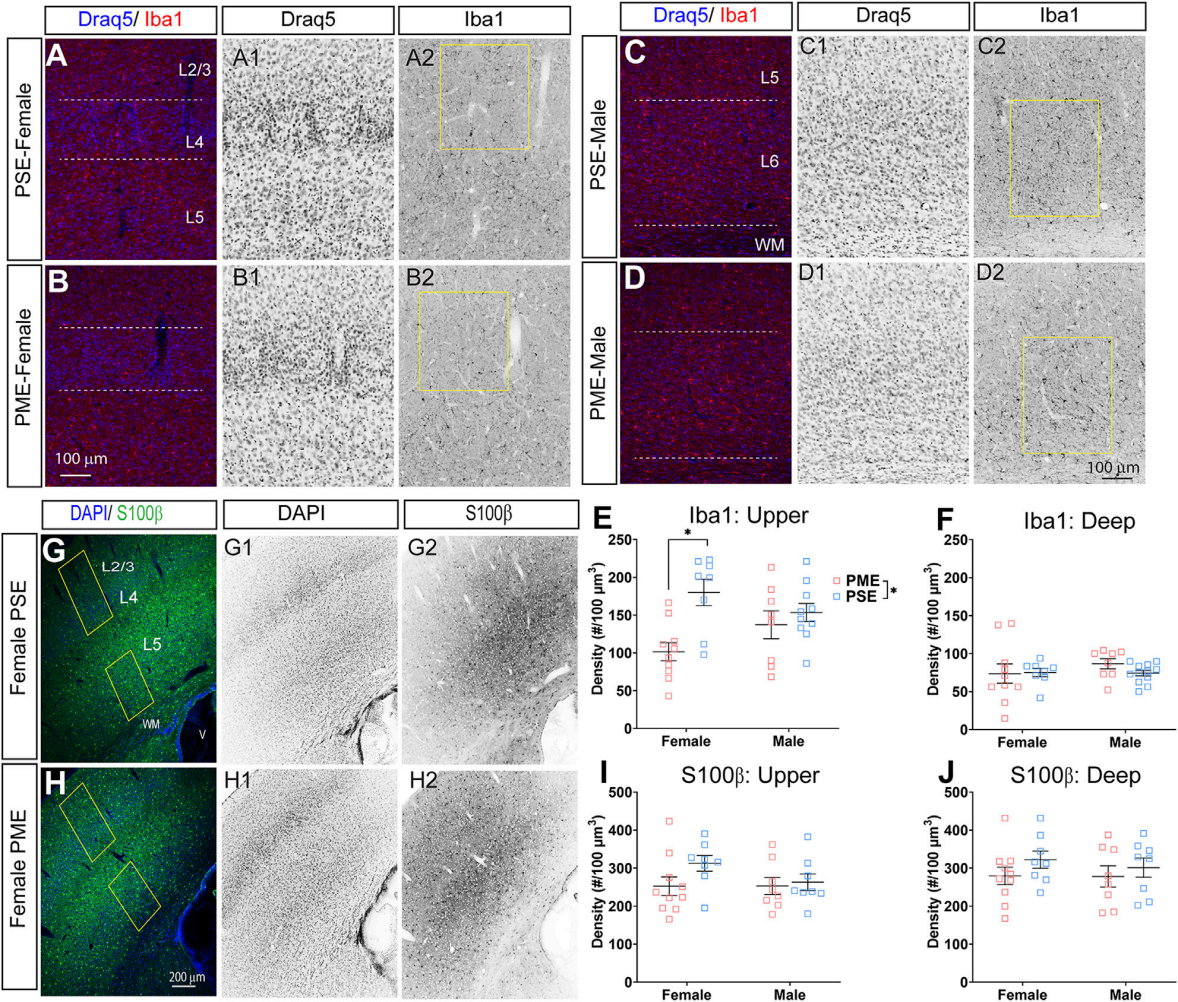
Neurochemical assessment of glia cell density. Representative slices of the S1 in a PME female **(A)** and a PSE female **(B)** demonstrating Draq5 [blue in **(A,B)**, isolated in **(A1,B1)**: marker of nuclei] and Iba1 [red in **(A,B)**, isolated in (**A2,B2)**: marker of microglia] in the upper layer. Similarly, representative slices of the S1 in a PME male **(C)** and a PSE female **(D)** demonstrating Draq5 [blue in **(C,D)**, isolated in **(C1,D1)**: marker of nuclei] and Iba1 [red in **(C,D)**, isolated in **(C2,D2)**: marker of microglia) in the upper layer. Yellow boxes represent the areas used for quantification. **(E)** Iba1+ cell density was significantly affected by PME in the upper layer (ANOVA: Exposure, *p* = 0.003; Interaction, *p* = 0.043) with PME female showing reduced densities in the upper layer compared to PSE females (*p* = 0.0014). **(F)** There was no impact of PME on Iba1+ density in the deep layer. Representative slices of the S1 in a PME female **(G)** and a PSE female **(H)** demonstrating DAPI [blue in **(D,E)**, isolated in (**G1,H1)**: marker of nuclei] and S100β [blue in **(D,E)**, isolated in (**G2,H2)**: marker of astrocytes]. Yellow boxes represent the areas used for quantification. There was no effect of PME on S100β in either the **(I)** upper or **(J)** deep layer. *n* = 9 (4M:5F) PME, 8 PSE (4M:4F). 1, 2 image per hemisphere per animal. *p < 0.05.

**FIGURE 14:**
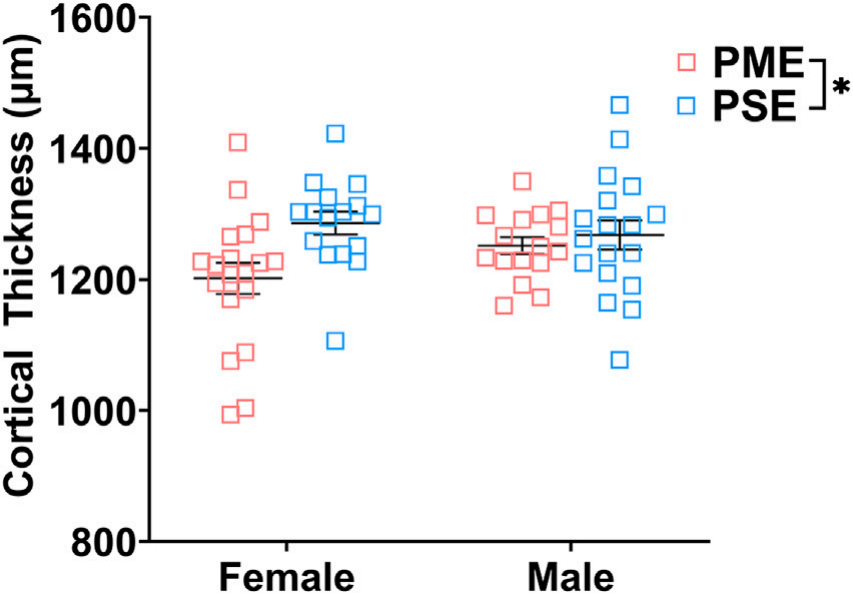
Cortical thickness of somatosensory cortex. PME significantly reduced cortical thickness of S1 (ANOVA: Interaction, *p* = 0.015). Although no significant main effect of sex or interactions with sex were present, this reduction in cortical thickness visually appears to be driven by PME females. *n* = 9 (4M:5F) PME, 8 PSE (4M:4F), one image per hemisphere, two brain section/animal.

Finally, we used microglia-specific calcium-binding protein, Iba1, and the calcium binding protein, S100β, that is primarily expressed in astrocytes and oligodendrocyte to assess microglia and astrocyte density in the S1 (See Figures 13A–D,G,H for representative images). There was a layer-specific effect of PME on Iba1 density as density was reduced in the upper layer (ANOVA: Exposure, F_(1,32)_ = 10.28, *p* = 0.003; Sex, F_(1,32)_ = 0.094, *p* = 0.76; Interaction, F_(1,32)_ = 4.45, *p* = 0.043; Figure 13E), with post hoc test indicating PME females specifically show reduced density (*p* = 0.0014). However, there were no group differences in Iba1 density in the deep layer of the S1 (ANOVA: Exposure, F_(1,34)_ = 0.45, *p* = 0.50; Sex, F_(1,34)_ = 0.56, *p* = 0.46; Interaction, F_(1,34)_ = 0.73, *p* = 0.40; Figure 13F). There was no effect of exposure present on S100β density within the upper (ANOVA: Exposure, F_(1,30)_ = 2.36, *p* = 0.13; Sex, F_(1,30)_ = 1.15, *p* = 0.29; Interaction, F_(1,30)_ = 1.20, *p* = 0.28; Figure 13I) or deep layer of S1 (ANOVA: Exposure, F_(1,30)_ = 1.75, *p* = 0.20; Sex, F_(1,30)_ = 0.20, *p* = 0.66; Interaction, F_(1,30)_ = 0.155, *p* = 0.70; Figure 13J). These results indicate PME reduces microglia densities in the upper layer of S1 with a greater impact in PME females but has minimal effects in the deep layer or in other glial cells. Lastly, in the process of assessing cell-type specific makers and synaptic markers in the S1, we also measured cortical thickness of the S1 in coronal slices and observed a significant reduction of cortical thickness as a result of PME which was primarily driven by PME females (ANOVA: Exposure, F_(1,65)_ = 6.22, *p* = 0.015; Sex, F_(1,65)_ = 0.617, *p* = 0.44; Interaction, F_(1,65)_ = 2.83, *p* = 0.097; Figure 14).

## Discussion

While the deleterious effects of opioids on the brain have canonically been described in regions associated with reward, the present study indicates prenatal exposure to opioids can impair S1 development. PME induced widespread changes to the proteomic and phosphoproteomic landscape of S1. These multi-omic changes were associated with several differences in excitatory and inhibitory synaptic development and cumulated in disrupted L2/3 pyramidal inhibitory neurotransmission. As these neurons represent a key node in the S1 microcircuit, excitatory/inhibitory imbalances in these cells could impair how inputs from L4 (the main recipient of thalamic information) are transferred to L5 pyramidal neurons (the main output neurons of the S1) which would likely lead to aberrant expression of sensorimotor behaviors. In addition to the alterations we have previously detailed in the motor cortex, these findings in the S1 could contribute to the impaired expression of the various developmental milestones in our PME offspring (14).

Impaired development of sensorimotor milestones and somatosensation have been widely observed in models of prenatal opioid exposure (11, 33–35). Similarly, deficits in S1 neurotransmission and morphology of S1 pyramidal cells that may contribute to these behaviors in opioid exposure models have also been described (35–38). Using a fentanyl exposure model where dams can freely consume fentanyl orally, Alipio et al. have found late adolescent mouse offspring exhibit numerous differences in the S1 including reduced excitatory transmission and increased inhibitory transmission in L5 pyramidal neurons (35). These L5 pyramidal neurons were also characterized by reduced dendritic branching and a smaller soma size which occurred alongside reduced expression of the neurotrophic receptor TrkB (35). In L2/3 neurons, they discovered fentanyl exposure impaired long term potentiation and increased the frequency of excitatory transmission in contrast to our null effects of PME on L2/3 mEPSC frequency (36). The present findings add to their work in several important ways. First, we provide the first comprehensive assessment of the proteomic and phosphoproteomic impact of PME on the S1 in both males and females. As the study of how prenatal opioid exposure impacts the S1 and somatosensory behaviors progresses, these data will provide an excellent, freely available resource and wealth of knowledge for any researcher to utilize. We identified numerous proteins and phosphopeptides of interest, and the enrichment analyses provide several molecular pathways that could serve to generate new hypotheses for future studies. Additionally, our synaptic marker analysis provides useful neurochemical context to supplement the electrophysiology findings in this present study and previous work (35, 36). Although we did not observe alterations in excitatory transmission, we have identified changes in inhibitory transmission likely reflecting postsynaptic changes in L2/3 neurons. Lastly, our cell-type marker analysis is the first assessment to demonstrate prenatal opioid exposure alters the density of glial cells in the S1 which could impact synaptogenesis and synaptic pruning.

It is worth noting that our model of PME and this previously discussed model of perinatal fentanyl exposure differ in many ways (39). Our mouse model seeks to recapitulate what is a growing clinical scenario: prenatal exposure to opioid agonists (e.g., methadone and buprenorphine) that treat OUD in pregnant women (12, 13) whereas Alipio et al. are modeling recreational fentanyl misuse in women (39). This is important as fentanyl and methadone differ in their potency for stimulating the mu opioid receptor (MOR; fentanyl>>methadone), their off target activities (e.g., physiologically relevant NMDA receptor antagonism for methadone), and their pharmacokinetic profiles (methadone classically has a very long half-life while fentanyl is relatively rapid) which undoubtably contribute to differences in their ability to cross the placenta and impact offspring development (40–43). Indeed, we and others have characterized the levels of methadone in offspring and determined that methadone tissue levels are quite high during the fetal period but drop to nearly undetectable levels in the first week of postnatal life (14, 44) leading to withdrawal in offspring around postnatal day 1 (14) which is a withdrawal time course that is similar to clinical observations (45). Alipio et al. report classic opioid withdrawal symptoms shortly after weaning (postnatal day 22) suggesting fentanyl passes through the breastmilk at high levels or offspring have access to freely consume the fentanyl solution alongside the dam as they mature, but an accompanying report of fentanyl tissue and/or plasma levels is not provided to lend any insights (39). Lastly, our recordings were completed in early adolescent mice (around 3, 4 weeks of age), a period when synaptogenesis, gliogenesis, and myelination are still rapidly occurring whereas Alipio et al. recorded from late adolescent mice (around 6–8 week old mice) when brain development is more stable (46). While conflicting findings could be due to any combination of these key variations between studies, we believe the present findings will act in conjunction with Alipio et al. to bolster the current understanding of how prenatal exposure to opioids can disrupt somatosensory functioning.

We initiated our exploration into the S1 by performing proteomics and phosphoproteomics of S1 bulk tissue from male and female PME and PSE offspring in attempt to identify processes or pathways which were uniquely affected by PME. For the global proteome, more proteins were identified as differentially abundant in males (83) than in females (52), yet the network of differentially abundant proteins in males yielded minimal enrichment (only 6 CCs were identified) while the female network generated dozens of enriched BPs and CCs. In females these BPs included “synaptic vesicle localization” and “synaptic vesicle clustering” and CCs included the “postsynapse,” “dendrite,” and “synaptic vesicle” indicating these terms are highly represented based on the differentially abundant proteins in PME females. Although the differential proteins and enrichment in the global proteome was quite distinct between males and females, the impact of PME on the phosphoproteome led to more similarities between males and females for the enrichment analyses. There were 38 CCs that were identified as enriched in both the male and female differential phosphopeptide network, and these CCs included “dendritic spine,” “presynaptic active zone,” “postsynaptic density” “excitatory synapse,” “glutamatergic synapse,” and “inhibitory synapse.” Similarly, 110 BPs were identified as enriched in both the male and female differential phosphopeptide network including processes such as “vesicle mediated transport in synapse,” “synapse organization,” “GABA secretion,” and “GABA transport.” The results from these enrichment analyses indicate there were many more alterations in proteins associated with these synaptic signaling processes and synaptic cellular locations than would be expected. Interestingly though, few proteins and phosphopeptides were identified as differentially abundant in both males and females suggesting PME has unique effects on the male and female proteome/phosphoproteome. However, while the individual proteins/phosphopeptides may differ, in many cases, the cumulative effect of these proteomic and phosphoproteomics effects still produced many similarities in GO and kinase enrichment. Nonetheless, these multi-omics findings served as an excellent source of hypothesis generating data as we later discovered several differences in GABAergic and glutamatergic synapses based on both anatomical and functional investigations.

There are some noteworthy limitations to bear in mind when considering this multi-omics analysis. First, although the differential protein/phosphopeptide expression in PME offspring indicated alterations in synaptic signaling were present, these enrichment analyses do not provide “directionality.” For instance, while the phosphopeptide abundances in PME offspring indicate the “GABA transport” BP is enriched, a GO enrichment analysis does not tell us that GABA transport is increased or decreased only that this BP is significantly represented given the list of differentially abundant phosphopeptides. This may explain why there was limited overlap in differentially expressed proteins/phosphopeptides, but more modest overlap in GO enrichment. Additionally, one-to-one comparisons between proteomics/phosphoproteomics data with synaptic marker or electrophysiology findings remain difficult as bulk S1 tissue was taken for the multi-omics analysis. Therefore, the quantified proteins/phosphopeptides may have originated in various S1 layers, glia cells, interneurons of S1, or even presynaptic inputs from other brain regions. This likely explains why differences in the density of synaptic markers were discovered in the immunostaining analysis, but these protein markers were not identified in the multi-omics analysis as differentially abundant.

How methadone induces these sex-dependent effects on the S1 remains to be investigated. Both *in vitro* and *in vivo* work has determined that the developing central nervous system expresses opioid receptors and opioid peptides during the prenatal period (47–50). During embryonic development, MOR agonists, including methadone, appear to inhibit growth, differentiation, and proliferation of neural and glial progenitor cells (48, 49). Additionally, the expression and functioning of the endogenous opioid system during early development is often transient meaning the effects of opioid exposure on the developing brain may differ considerably from the effects of opioids on an adult brain (48). Therefore, exposure to the exogenous opioid methadone during this critical period of embryonic neurodevelopment could disrupt MOR-mediated signaling leading to a lasting disruption in S1 neuronal development and synaptogenesis. Although we have previously identified sex-dependent effects in reward-related behavior in this PME model (51), the sex differences discovered across various modalities of data collected in the present study add an additional layer of complexity that was unexpected. There is evidence for cross-talk between estrogen signaling and MOR expression (52, 53). As hypothalamic–pituitary–adrenal axis dysfunction is observed in opioid exposed offspring (54, 55), it is possible PME also disrupts the hypothalamic-pituitary-gonadal axis altering steroidal hormones concentrations or their receptors during the perinatal period when the brain is uniquely sensitive to the enduring effects of these hormones (56) which may contribute to the observed sex differences in opioid exposed offspring. The mechanisms underlying the sex-dependent effects of PME on offspring behavioral and brain development will require further investigation.

In summary, our findings indicate PME induces prominent disruptions in the S1 in a sex-dependent manner. Dozens of proteins and phosphopeptides display differential abundance in PME offspring with functional enrichment in several relevant pathways including those related to synaptic transmission. PME offspring also exhibit layer-dependent differences in GABAergic markers, glutamatergic markers, and microglia density. Lastly, PME has functional consequences on S1 neurotransmission as L2/3 pyramidal neurons exhibit disrupted inhibitory transmission. These findings suggest deficits in sensorimotor development observed in models of prenatal opioid exposure may result from persistent neuroadaptations induced by opioid exposure during fetal development of the S1.

## Data Availability

The raw data supporting the conclusions of this article will be made available by the authors without undue reservation. All supplementary files and raw data can be found at https://github.com/gggrecco/S1-Omics.
